# Dietary Carbohydrate as Glycemic Load, Not Fat, Coupled with *Genetic Permissiveness* Favoring Rapid Growth and Extra Calories, Dictate Metabolic Syndrome and Diabetes Induction in Nile Rats (*Arvicanthis niloticus*)

**DOI:** 10.3390/nu14153064

**Published:** 2022-07-26

**Authors:** Avinaash Subramaniam, Bumjoon Park, Domenick Raphael, Michelle Landstrom, K. C. Hayes

**Affiliations:** Department of Biology, Brandeis University, Waltham, MA 02453, USA; avinaash1381@gmail.com (A.S.); bumjoon95@gmail.com (B.P.); domenick.raphael@gmail.com (D.R.); landstrm@gmail.com (M.L.)

**Keywords:** macronutrient ratio, type 2 diabetes, high-carbohydrate diet, high-fat diet, glycemic load

## Abstract

Objective: Whether dietary carbohydrate (CHO) or fat is more involved in type 2 diabetes (T2DM) induction uncomplicated by dietary fiber was addressed in a spontaneous diabetic model, the diurnal Nile rat that mimics the human condition. Methods: A total of 138 male Nile rats were fed plant-based and animal-based saturated fat where 10% energy as CHO and fat were exchanged across 5 diets keeping protein constant, from 70:10:20 to 20:60:20 as CHO:fat:protein %energy. Diabetes induction was analyzed by: 1. diet composition, i.e., CHO:fat ratio, to study the impact of diet; 2. *quintiles of average caloric intake per day* to study the impact of calories; 3. *quintiles* of diabetes *severity* to study the epigenetic impact on diabetes *resistance*. Results: High glycemic load (GLoad) was most problematic if coupled with high caloric consumption. Diabetes *severity* highlighted rapid growth and caloric intake as likely epigenetic factors distorting glucose metabolism. The largest weanling rats ate more, grew faster, and developed more diabetes when the dietary GLoad exceeded their gene-based metabolic capacity for glucose disposal. Diabetes risk increased for *susceptible* rats when energy intake exceeded 26 kcal/day and the GLoad was >175/2000 kcal of diet and when the diet provided >57% energy as CHO. Most *resistant* rats ate <25 kcal/day independent of the CHO:fat diet ratio or the GLoad adjusted to body size. Conclusion: Beyond the CHO:fat ratio and GLoad, neither the type of fat nor the dietary polyunsaturated/saturated fatty acid (P/S) ratio had a significant impact, suggesting *genetic permissiveness* affecting caloric and glucose intake and glucose disposition were key to modulating Nile rat diabetes. Fat became protective by limiting GLoad when it contributed >40% energy and displaced CHO to <50% energy, thereby decreasing the number of diabetic rats and diabetes severity.

## 1. Introduction

Type 2 diabetes mellitus (T2DM) and metabolic syndrome (MetS) represent metabolic disturbances that are steadily rising in prevalence globally. MetS is characterized by insulin resistance with hyperinsulinemia, hypertension, decreased high-density lipoprotein (HDL), increased visceral adiposity, and fatty liver with increased plasma triglycerides (TG), which can lead to rising blood glucose, hyperglycemia and T2DM if sustained [1,2,3]. A heated debate with much-contradicting evidence exists to explain how macronutrient content (i.e., CHO, fat, and protein) and the quality of said macronutrients (i.e., refined vs. complex CHO, fiber, types of fatty acids, animal vs. plant-based fat, and protein sources) affect the onset and progression of T2DM. While certain studies indicate that the more carbohydrates one consumes, the more likely one is to develop diabetes, other studies have suggested that dietary fats are implicated in diabetes development [4].

Few conclusive findings illustrate how different types of fats, such as saturated fat and unsaturated fat, comprising monounsaturated fatty acids and/or polyunsaturated fatty acids, affect the outcome of diabetes. Several studies have investigated the response in blood glucose among those who follow a particular diet in order to reduce the *severity* of T2DM and MetS. However, insufficient research indicates how specific diet compositions modulate the course of T2DM induction. This is partly because the macronutrient matrix and various combinations of CHO: fat:protein %energy mixes are extremely difficult to control in clinical trials of appreciable duration. While epidemiological data suggest a major impact of dietary fat and its association with T2DM in adults [5,6,7], preclinical data where nutritional parameters have been manipulated during growth suggest that CHO, not fat, is responsible for most of the risk [1]. Studies with humans have also consistently shown that CHO content and GLoad are strongly related to adult-onset T2DM and MetS [8,9,10,11,12].

In effect, it is generally accepted that CHO and the dietary GLoad are the two primary dietary contributors to the increase in blood glucose and eventual diabetes. Restricting CHO, as opposed to restricting fat, produces the greatest reduction in blood glucose, be it postprandially or with other measures of chronic glucose overload, including HbA1c. Refined CHO, in particular, produces more harmful metabolic effects than equivalent calories from saturated fat [4,12,13,14,15,16,17,18]. Thus, one of the issues in MetS and T2DM in humans, or any species, is accounting for the source and metabolism of calories dictated by the composition of the diet consumed [19,20,21]. The pressing quandary is whether diabetes risk—with protein calories kept constant—is more aligned with CHO calories (as GLoad) or fat calories (as a bulk or adverse source of saturated fatty acids). To address this problem, a suitable model that reflects the human disease is required. The Nile rat, also known as the African grass rat (*Arvicanthis niloticus*), has been developed as a unique animal model of diet-induced diabetes (see more details in Materials and Methods) [1,22]. Disruption of the circadian rhythm induced by laboratory housing conditions is likely involved because adhering to natural light/dark cycles in captivity prevents high-energy diet-induced diabetes in a closely related model, the Israeli sand rat [23,24].

It is unclear, however, whether the hiCHO, i.e., 70%, 60% and 50% energy as CHO, or possibly a low dietary fat content that limits essential fatty acids (EFAs) was a key factor in previous Nile rat and sand rat studies. Furthermore, the relative absence of polyunsaturated fatty acids and EFAs may also be a factor because diacylglcerol, ceramide, and related structural lipids as cell signals have been implicated in insulin resistance and as a trigger for T2DM [25,26,27,28]. Diacylglycerol’s composition, in part, reflects the triglyceride (TG) molecular species consumed in the diet by the host [29,30], with plant-based TGs being more desirable as a better source of linoleic acid (18:2(n-6)) than animal-based TGs, where more saturated fatty acids mean minimal 18:2(n-6). This is supported by the evidence that 18:2(n-6) consumption reportedly protects against T2DM [31,32,33,34].

Finally, to date, no meaningful epidemiological studies on T2DM have focused on dietary intake in children less than 20 years of age or in young adults when the risk of T2DM first occurs.

Previous Nile rat data have detailed how rapidly growing pups that ate the most food (as kcal/day), including a high glycemic index (GI) and thus, a high cumulative GLoad (cumGLoad), developed the most diabetes. Additionally, their susceptibility to diabetes was strongly related to apparent *genetic permissiveness*, coupled with the cumGL consumed during the course of the experiment [35]. Furthermore, fat quality or fat calories did not appear to have much effect within the normal range of intake, in keeping with the human condition [12,36]. Therefore, given this background, we conducted a controlled experiment to investigate the effect of diet composition, i.e., macronutrient ratios, on the onset and progression of T2DM by altering CHO and fat while keeping protein constant.

## 2. Materials and Methods

### 2.1. Animal Model (Nile Rat, Arvicanthis niloticus)

The Nile rat, a diurnal North African desert rodent, develops ‘spontaneous’ diabetes when housed in captivity and fed typical commercial mouse/rat chow low in fiber, i.e., Lab Diet 5008; 65:8:27 as CHO:fat:protein %energy with 3% fiber. Furthermore, semi-purified hiCHO-low fat diets devoid of fiber, for example, 70:10:20 CHO:fat:protein %energy, actually accelerate and amplify diabetes, while supplementation of certain fibers or polyphenols deters it [1,35,37]. In essence, the young Nile rat is exquisitely prone to T2DM during growth when fed a hiCHO diet in the absence of fiber. Many data detail how rapidly growing male pups that eat the most food containing a high Gload develop the most diabetes. Additionally, susceptibility to diabetes is strongly related to apparent *genetic permissiveness*, coupled with the consumed cumGL during the course of the experiment [1,35,38,39].

All experiments and procedures were approved by the Brandeis University Institutional Animal Care and Use Committee (approval code: IACUC #14005).

### 2.2. Semipurified Diets

To address unresolved issues pertaining to diet composition involving CHO and fat, including that of human epidemiological studies, semipurified diets were formulated and mixed onsite to be fed to growing rats. In the current experiments, dietary CHO was systematically replaced with fat in increments of 10% energy. Hence, the diets ranged from 70:10 CHO:fat to 20:60 CHO:fat with protein held constant at 20% energy, e.g., 70:10:20 to 20:60:20 for the CHO:fat:protein %energy, respectively (see Table S1 and reference [40]). Since CHO and fat were the dietary variables being exchanged, protein was kept constant to ensure that no harmful or beneficial effects resulted from an altered protein component that might have complicated the interpretation to render the study inconclusive.

#### Fatty Acid Profiles

Epidemiological studies imply that animal-based saturated fats, in particular, coupled with a high GLoad from refined CHO, increase the risk of T2DM in adults while polyunsaturated fatty acids protect against diabetes [10,31,34,41,42]. Therefore, the diets were designed to alter these CHO:fat compositions to study their impact on diabetes by altering the macronutrient ratio. Since saturated fat has been implicated as a potential risk factor in T2DM epidemiology in humans and animals [34,41,42,43], two sources of saturated fats with varying hypercholesterolemic effects were compared for T2DM induction [44]. The fat sources were a palm kernel oil blend (PKOB) and the American fat blend (AFB). The PKOB blend was plant-based, consisting of blended palm kernel oil, canola oil and soybean oil, rich in medium-chain triglyceride (MCT) fatty acids, i.e., 8:0–12:0. Canola oil and soybean oil were added to balance the saturated:monounsaturated:polyunsaturated fatty acid (S:M:P) ratio to 1:1:1 with a P/S ratio of 1.0, which is ideal metabolically for lipoprotein metabolism [45,46]. AFB was designed based on animal saturated fats with a relatively low P/S ratio of 0.35, typical of fat in the American diet [47,48,49,50]. AFB consisted of butter, tallow, lard and soybean oil, which together featured long-chain fatty acids rich in palmitic (16:0) and stearic (18:0) fatty acids. The S:M:P %energy ratio of the AFB was 9:8:3, similar to that in the American diet. See Table S2 for details on fatty acid profiles.

### 2.3. Experimental Design

To complete the two experiments, 138 male Nile rats from the Foster Biomedical Research Labs breeding colony at Brandeis University were separated at weaning. Rat pups were 3 weeks old with random body weight (RBW) initially averaging 32 g, obtained from breeders feeding the mouse LabDiet 5008. Pups were fed the experimental diets (Table S1) for 1 week before food intake was recorded to allow the rats to acclimate to the diet. All Nile rats were placed in individual small-sized cages (13 × 7 × 6 in) in an air-conditioned room with a 12-h light/dark cycle, temperatures of 68–72 °F, and humidity of 40–60%. All the Nile rats were wild-type, i.e., randomly bred and not a product of inbreeding. All experiments and procedures were approved by the Institutional Animal Care and Use Committee (IACUC).

For Experiment 1 (PKOB), 69 male Nile rats were divided into 5 dietary groups of CHO:fat:protein %energy based on PKOB fat as follows: 70:10:20, 60:20:20, 50:30:20, 40:40:20, 20:60:20. Each dietary group had a sample size of *n* = 13–14. Diets were fed 3 times a week. Experiment 2 (AFB) was identical to Experiment 1, but the fat was AFB.

### 2.4. Calculations, Measurements and Data Analysis

#### 2.4.1. Body Weight, Growth and BMI

Body weight (BW) in grams was measured at the start and at weeks 6 and 10 for both experiments. Growth as BW gained per day was measured (in g/day) throughout the 10-week study. BMI, on the other hand, was calculated by dividing terminal fasting BW, in kg, by body length squared (as m^2^) to establish a BMI with standard units in kg/m^2^.

#### 2.4.2. Food (Caloric) and Water Intake

Once the pups were separated, their semipurified diet was weighed and rolled into a medium-sized bolus, then placed in the wire food bin on top of the cage. Usually, powdered rat chow was pressed onto the food surface for the first week to acclimate the pups to their new semipurified diet. The diet was changed on Monday, Wednesday, and Friday, at which time the remaining diet was weighed to determine consumption, and the amount of fresh food was adjusted accordingly. The total grams of food obtained for the week were summed and then divided by 7 for the g/day for that week, which was then converted to kcal/day based on the diet composition (Table S1). In the 9th week of the study, one week before necropsy, new water bottles were filled, and the weight was recorded after a week to determine the water consumed (g = mL) at the 10th week.

#### 2.4.3. Food Efficiency

Food efficiency was calculated by dividing BW gain per day, in g/day, by caloric intake (daily food intake in kcal/day) and multiplying the result by 1000. Results represent the grams of BW gained per 1000 kcal consumed. Thus, greater food efficiency represents greater weight gained per calorie.

#### 2.4.4. Glycemic Index

Dietary GI was calculated as defined in the literature [1,9,36,39,51,52] based on the respective sources of CHO in each diet, i.e., dextrose (1.0), cornstarch (0.77), and sucrose (0.65).

#### 2.4.5. Glycemic Load

After obtaining the GI of the various dietary ingredients published in the literature and summarized online (verywellhealth.com/glycemic-index, accessed 10 July 2022), the Gload was calculated for each diet with the CHO content in each ingredient using the following formula:

GLoad/kg of diet as described in Tables S1 and S2 = (sum of net CHO in g for each ingredient) × (GI of that respective CHO/kg diet)

Amounts were converted to GLoad per 2000 kcal to approximate a comparative daily human equivalent, which is typically 75 to 125/2000 kcal.

#### 2.4.6. Blood Glucose

Blood glucose was measured in 50/50 O_2_/CO_2_ anesthetized rats from a drop of tail blood, obtained by lancet puncture of the lateral tail vein and recorded with a Bayer Next Contour glucometer (Bayer Co., Elhart, IN, USA).

##### Random Blood Glucose (RBG)

RBG was assessed in nonfasted rats between 10 a.m.–2 p.m. on non-feeding days. In healthy rats, postprandial RBG rises to 100–150 mg/dL and then typically returns to <75 mg/dL in 1 h. RBG > 75 mg/dL is considered the breakpoint for prediabetes-diabetes initiation in Nile rats fed commercial mouse/rat chows, whereas prediabetes RBG stays above >75–100 mg/dL. The rationale for using the RBG, not fasting glucose, for diabetes assessment has been presented previously [1,35,38,39,49,53,54,55].

##### Diabetes Severity and Susceptibility

The severity of diabetes was characterized by RBG rising above 75 mg/dL, i.e., increasing progressively over time until rats were severely diabetic and insulin-deficient with RBG in the 400–600+ mg/dL range. Susceptibility defines the dietary impact on diabetes for any given group of rats. This was expressed as the ratio between diabetic (*susceptible*) Nile rats and the total population, comprising both *susceptible* and *resistant* rats fed that particular diet. Susceptibility generally ranges from 0–10% *susceptible* when rats are fed a high-fiber, low-GLoad diet, to >80% *susceptible*, when rats are fed a diabetogenic hiCHO, fiber-free diet [1,35].

The rate at which the pool of *susceptible* rats increases with diet challenge is considered an expression of their *genetic permissiveness* for developing T2DM when consuming that diet [1,35,39]. Thus, depending on the breeding stock, about 50% of the pups are *resistant* to diabetes induction by diet, while the other 50% become diabetic in the 10 weeks following weaning and are considered *susceptible* to induction under the right dietary insult. This genetic or epigenetic aspect of susceptibility was discovered in previous studies by feeding all Nile rats one diet and following their RBG over 8–10 weeks from weaning (between 3–13 weeks of age) [1,22,35,38,56]. By altering the macronutrient composition ratios, the aforementioned semipurified diets were designed to either increase or decrease the *resistance* or *susceptibility* to diabetes to determine which dietary component exerts the most impact on diabetes.

##### Fasting Blood Glucose (FBG)

Healthy FBG in young growing Nile rats ranges between 40 and 60 mg/dL in young rats. FBG was measured at 9–10 a.m. after 16 h overnight food deprivation at the start (0-time) of the oral glucose tolerance test (OGTT).

##### OGTT

Rats were fasted for 16 h overnight to start the oral glucose tolerance test. After assessment of BW and fasting blood glucose, rats were dosed with 1.75 g/kg BW of dextrose solution (10.5 g in 6 mL distilled water) by means of oral gavage, and blood glucose was typically assessed at 30 and 60 min by tail bleed after gavage because this truncated curve was sufficient to determine the relative *severity* of T2DM between Nile rats for the diet being tested. Although a somewhat elaborate technique, the OGTT is the most sensitive assay one can apply.

#### 2.4.7. Organ Weights

Organs were weighed after excision, and their weight (in g) was divided by terminal BW to obtain organ weights as a percent of BW (%BW). The carcass weight (as %BW) was determined by weighing lean body mass (after exsanguination and excision of all organs except the brain) and dividing it by terminal fasting BW.

#### 2.4.8. Plasma Triglycerides (TG) and Total Cholesterol (TC)

TG and TC were determined spectrophotometrically using Infinity kits (Thermo Fisher Scientific Inc., Middletown, VA, USA, TG ref # TR22421, TC ref # TR13421).

#### 2.4.9. Statistical Analyses

Statistical analyses were performed using SPSS version 27 (SPSS, Inc., Chicago, IL, USA). One-way or two-way analysis of variance (ANOVA) were conducted where appropriate to the study design.

#### 2.4.10. Ethics Statement

All experiments and procedures were approved by the Brandeis University Institutional Animal Care and Use Committee (approval code: IACUC #14005).

### 2.5. Data Analysis

The data from both current experiments were analyzed in three ways:

(1) By assigned diet groups to determine if any particular diet composition or the macronutrient CHO:fat ratio might explain the majority of the diabetes induced.
(2) By quintiles of caloric intake. The data were reassessed by quintiles based on average kcal/day consumed from lowest (Q1_kcal_) to highest (Q5_kcal_) to determine if kcal/day intake, regardless of macronutrient ratio, would be more revealing than the sort by diet composition that identified rats as resistant (<75 mg/dL) or susceptible (>75 mg/dL) subgroups.
(3) By quintiles of 10-week RBG. The RBG for rats in each experiment were pooled and sorted into quintiles (Q1_RBG_ to Q5_RBG_) from lowest to highest to determine whether RBG would expose additional aspects of diabetes established following the first two analyses.

## 3. Results

### 3.1. Analysis by Diet Composition

#### 3.1.1. Diet Composition Affects T2DM Induction and Severity

**Figure 1:**
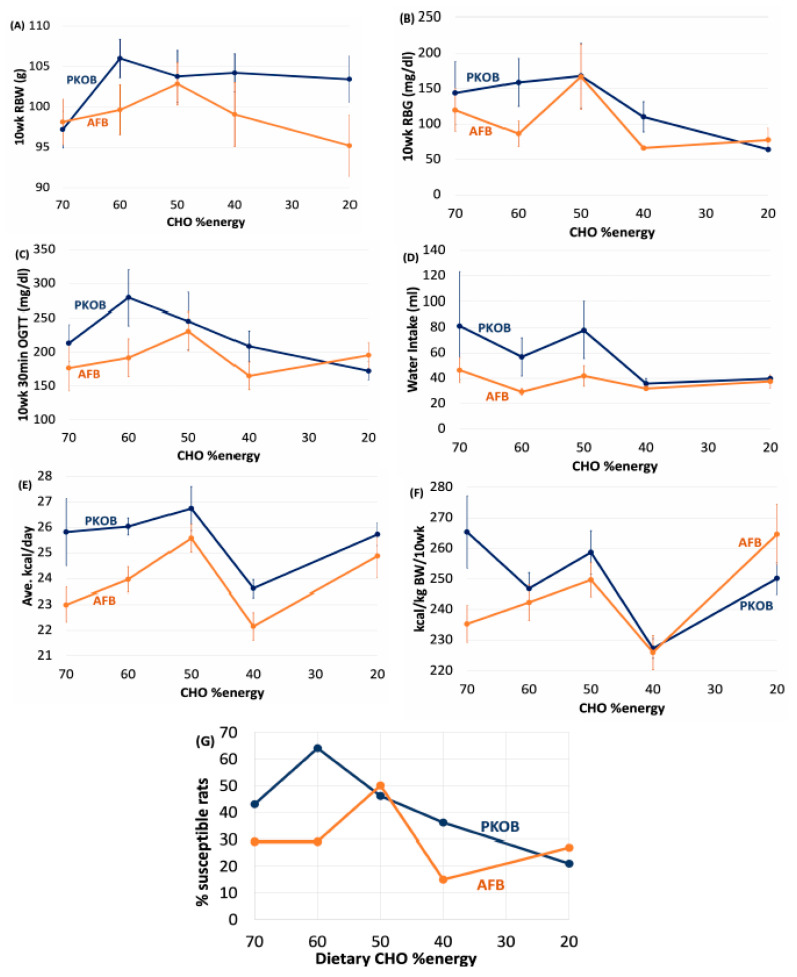
Clinical results for diabetes induction are depicted for all Nile rats (*n* = 14 rats per CHO:fat ratio) based on the dietary CHO %energy designated on the X-axis starting at 70, 60, 50, 40, and 20% energy with corresponding fat increments at 10, 20, 30, 40 and 60% fat energy, respectively, for PKOB (blue) and AFB (orange). (**A**) PKOB tended to produce greater RBWs than AFB. (**B**) RBG revealed more diabetes with PKOB, i.e., 42% of rats for PKOB vs. 29% for the AFB. (**C**) The 30′ OGTT confirmed that diabetes was more severe with PKOB than AFB, especially at the two highest intakes of CHO. (**D**) Water consumption was greater for rats fed PKOB than AFB, but only for the 3 highest intakes of CHO at 70, 60, and 50% energy linked to diabetes severity. (**E**) Kcal/day was generally higher for PKOB and significant at 70 and 60 CHO %energy. (**F**) Once adjusted for BW, kcal/day was similar for all CHO intakes for both fats, except for 70% energy from CHO, where PKOB was greater than AFB. (**G**) PKOB induced a greater percentage of *susceptible* rats at 70, 60, and 40 CHO %energy, reflecting the greater extent of diabetes for PKOB (45% more) than AFB in general, as depicted in (**B**) above. The AFB with 50% energy from CHO also induced unique peaks in terms of RBG and *%susceptible* rats. In summary, these clinical data indicate that PKOB rats weighed more, consumed more calories and drank more water, with the most diabetes. This occurred at the 3 greatest hiCHO intakes for both fat types (see Tables S3 and S4 for details).

Comparing the 5 diet groups in both experiments demonstrated that decreasing the CHO:fat ratio caused the incidence of diabetes (indicated by RBG and 30′ OGTT) to decline gradually across the diet groups, i.e., as the percentage of fat energy incrementally replaced CHO in both PKOB and AFB diets. Overall, diabetes developed in 42% of PKOB fed rats (29/69 rats), but only in 29% (20/69 rats) of AFB-fed rats (45% fewer) (Tables S3 and S4; Figure 1G). Most of the difference was attributed to diets that provided the most CHO %energy, i.e., 70, 60, and 50 CHO %energy. Diabetes in PKOB-fed rats peaked at 64% susceptible within the 60:20:20 diet group, while diabetes peaked at 50% in the AFB-fed rats in the 50:30:20 diet group. The decline in susceptible rats within each diet group was more abrupt as fat replaced CHO (Tables S3 and S4; Figure 1G). The severity of diabetes followed the same pattern, i.e., the more diabetes present in a diet group, the greater the *severity* of their diabetes (RBG > 75 mg/dL). Additionally, fasting blood glucose (FBG) even tended to increase slightly after 10 weeks for rats fed the most fat (Tables S3 and S4). Thus, T2DM tended to be greater in rats fed the most CHO with the greatest GLoads at 70, 60 and 50 CHO %energy for both PKOB and AFB and was least evident at the lowest-CHO, greatest-fat intakes (40:40:20, 20:60:20) that also provided the lowest Gloads (Tables S3 and S4; Figure 1). The trend for decreased diabetes with increasing dietary fat intake was evidenced by decreases in Gload, RBG, the 30′ OGTT at 10 weeks, and water intake. This was most apparent for rats fed PKOB diets, where the diabetes was more extensive. In addition, plasma TG was elevated by hiCHO diets at the end of the study, especially by the 60:20:20 and 50:30:20 diets (Tables S3 and S4).

#### 3.1.2. Diet Effect on BW and Caloric Intake

**Figure 2:**
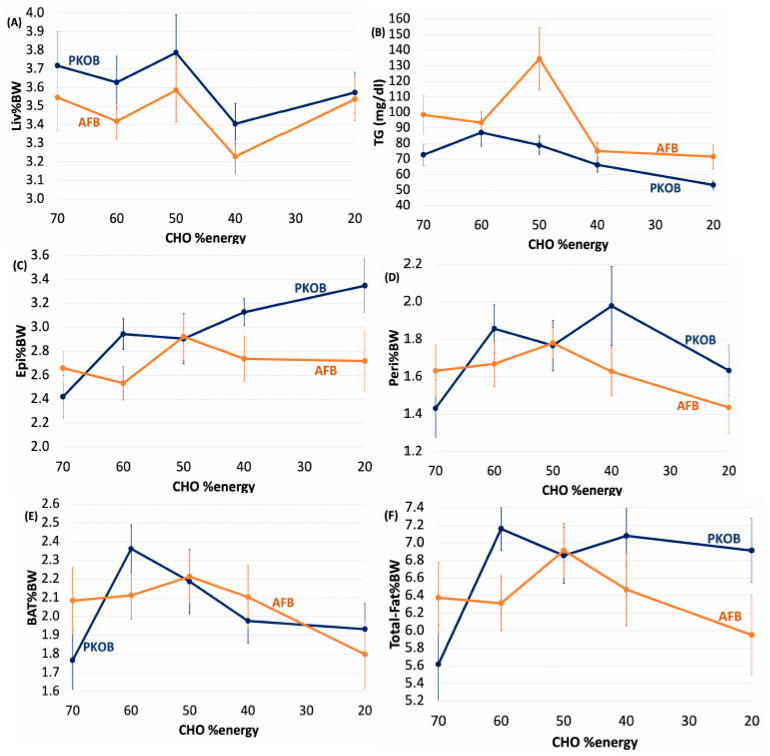
Necropsy findings for organ weights and plasma TG associated with the CHO:fat ratio across the five diet groups for both PKOB (blue) and AFB (orange). The data follow a pattern similar to that observed in the clinical data in Figure 1. (**A**) PKOB increased liver %BW, and the increase was greater with hiCHO. (**B**) TG was actually greater for AFB than for PKOB, and it peaked with 50% energy as CHO. This trend and peak mirrored that of RBG (Figure 1B) and *%susceptible* rats (Figure 1G). (**C**) Epididymal fat as %BW (Epi%BW) increased along with fat intake (with CHO intake decreasing) in rats fed PKOB. This trend is converse to the trends we see with TG and RBG, which increases when CHO is high. (**D**) PKOB increased the perirenal fat pad as %BW (Peri%BW) compared to the AFB. (**E**) BAT%BW increased with hiCHO %energy, except for 70% energy as CHO, suggesting a general compensatory attempt to reverse diabetes by increasing saturated fatty acid energy catabolism. (**F**) Total body fat as %BW was greater for PKOB, and it increased as the %energy from dietary fat increased, but not for AFB (see Tables S3 and S4 for added details).

**Figure 3:**
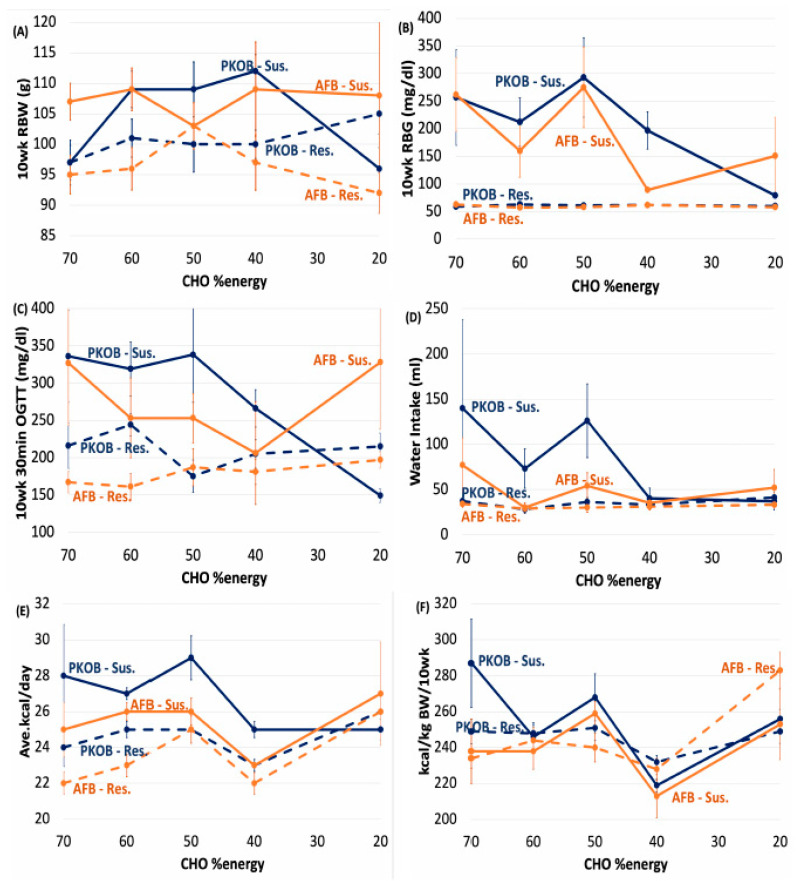
Clinical data for all rats reflect diabetes induction by PKOB (blue) and AFB (orange) as a function of the CHO:fat ratio on the X-axis. *Genetic permissiveness* was expressed as rats *resistant* (dashed lines) and *susceptible* (solid lines) to diabetes. (**A**) RBW for *resistant* rats was less than that for *susceptible* rats for both PKOB and AFB. The weight difference was most striking for AFB, where *susceptible* rats weighed substantially more than *resistant* rats. (**B**) RBG for all *resistant* rats was normal, around 50 mg/dL, whereas *susceptible* groups fed PKOB expressed more diabetes than *susceptible* rats fed AFB (42% vs. 29%), which was in keeping with overall more diabetes in PKOB rats overall. (**C**) The 30′ OGTT revealed the most diabetes (>175 mg/dL) at 70, 60, 50 and 40 CHO %energy, with less diabetes in *resistant* rats fed AFB than PKOB at 70 and 60 CHO %energy intakes. In general, the 30′ OGTT is more sensitive to detecting early diabetes than RBG. (**D**) Water intake was normal for both groups of *resistant* rats, but was most elevated in *susceptible* rats fed PKOB at 70, 60 and 50 CHO %energy, when the diabetes was most pronounced. (**E**) Average kcal/day intake was consistently greater for *susceptible* rats fed 70, 60, 50 and 40 CHO %energy compared to *resistant* rats for both fats. However, at the greatest fat intake, i.e., 20% CHO, 60% fat, caloric intake was similar for all rats. In general, *susceptible* rats consumed more than 25 kcal/d, while *resistant* rats ate 22–25 kcal/d. (**F**) When caloric intake was adjusted for BW, PKOB rats at 70 CHO %energy ate more calories than all other rats (except for a lone *resistant* outlier fed AFB at 20 CHO %energy as CHO). Differences between PKOB and AFB decreased as CHO %energy decreased and fat intake increased (see Tables S5 and S6 for details).

**Figure 4:**
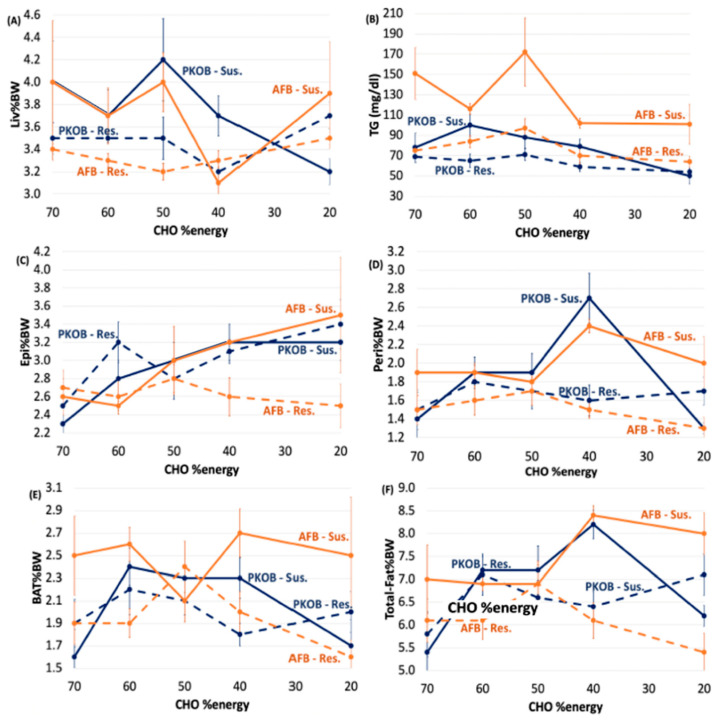
Differences in organ weights and plasma TG are depicted for rats that are *resistant* (dashed lines) and *susceptible* (solid lines) to diabetes. (**A**) Liver as %BW was greater in *susceptible* rats, and it was lowest at the highest fat intake where the least diabetes occurred. Liv%BW. (**B**) TG was greater in *susceptible* rats, especially those fed AFB, where increases at 70, 60 and 50 CHO %energy suggests a rising atherogenic risk associated with a lower 18:2(n-6) intake and P/S ratio of only 0.35 in the AFB. (**C**) Epididymal fat increased as the dietary CHO %energy decreased, and fat %energy increased, except for the AFB *resistant* rats, where it was greatly decreased by high fat. (**D**) Perirenal fat was the most energy-sensitive white adipose tissue. The spike in Peri%BW at the CHO:fat ratio of 40:40 %energy suggests that this white adipose tissue deposit may inversely reflect food intake by an undisclosed mechanism. For example, compare with Figure 3F, where all *susceptible* rats ate fewer BW-adjusted calories at 40% from CHO. (**E**) Brown Adipose Tissue (BAT) tended to be greater in *susceptible* rats fed AFB rats; BAT for both groups of *susceptible* rats generally was greater than that for comparable *resistant* rats. (**F**) Total fat reflected the perirenal fat response, i.e., *susceptible* rats in both groups had more total fat than the *resistant* rats, especially at 40% CHO %energy (see Tables S5 and S6 for details).

Neither BW nor caloric intake were affected much by diet composition when diet groups were compared (unsplit for diabetes) within or between the two fat studies (Tables S3 and S4; Figure 1 and Figure 2). However, when rats were split as either *resistant* or *susceptible* to diabetes, the *susceptible* rats were typically larger (Tables S5 and S6; Figure 3 and Figure 4). BW tended to follow caloric intake for PKOB, but not so much for AFB, once caloric intake was adjusted for body size (Figure 1F and Figure 3F; Tables S5 and S6). This suggests that food intake and T2DM were driven by genetic factor(s) that affected growth rate rather than by diet composition per se.

#### 3.1.3. Diet Effect on Food Efficiency

No clear pattern was discerned for food efficiency as a function of diet (Tables S3 and S4), which implies that any differences in food intake or growth rate (as BW gain per day) on diabetes expression were not simply reflecting a subpopulation of rats, e.g., a susceptible rat population that was more efficient in their use of calories for growth than another, more resistant population (Tables S5 and S6).

#### 3.1.4. Diet Effect on Water Intake

Rats consuming the most CHO at 70, 60 and 50% energy tended to drink more water, corresponding to their greater incidence of diabetes. Thus, per previous observation, the diabetic (*susceptible*) rats consuming diets with greater GLoads, especially PKOB-based diets, drank more water than the nondiabetic (*resistant*) rats fed the same diet (Tables S5 and S6; Figure 1D and Figure 3D). Not surprisingly, polydipsia did not pertain to the two diets providing the greatest fat intakes as a group, i.e., 40% and 60% fat energy, which coincided with their reduced incidence and *severity* of diabetes. This presents further evidence that high dietary fat was protective and that hiCHO loads exacerbated diabetes in diets with PKOB. Consequently, increasing water intake can be considered a sensitive measure of the T2DM progression in the model (Tables S5 and S6).

#### 3.1.5. Diet Effect on OGTT

Similar to the RBG, the OGTT typically was more responsive in rats fed the three diets with the greatest amount of CHO and Gload, especially for PKOB diets. By contrast, both the PKOB and AFB diets with the most fat (20:60:20 %energy) appeared somewhat protective against an elevated 30′ timepoint in the OGTT (Tables S3 and S4). Additionally, not surprisingly, the 30′ timepoint of the OGTT, like RBG, was substantially elevated in *susceptible* rats in both experiments compared to *resistant* rats, in keeping with the RBG, Liv%BW, and plasma TC results (Tables S5 and S6).

#### 3.1.6. Diet Effect on Organ Weights

Generally, organ weights for all Nile rats as a group were relatively unaffected by diet composition, although total body fat %BW was typically lower for rats fed the AFB diet (Tables S3–S6; Figure 2 and Figure 4). The livers at necropsy were typically enlarged in *susceptible* rats fed either type of fat, with PKOB associated with a greater number of diabetic rats (Tables S5 and S6).

The cecum (Cec%BW) tended to be larger for *susceptible* rats fed the 70:10:20 %energy diet formulated with PKOB, implying that the microbiome was altered most by the greatest GLoad. Decreased brown adipose tissue (BAT) as %BW suggests that ketosis and fatty acid oxidation were accelerated in *susceptible* rats fed the 70:10:20 diet containing PKOB. BMI tended to be greater in *susceptible* rats fed PKOB, complementing their greater kcal intake and BW gain and a greater percentage of body fat, in general (Figure 1, Figure 2, Figure 3 and Figure 4; Tables S3–S6).

#### 3.1.7. Diet Effect on Plasma Lipids

When dietary fat content reached 40% energy or more in both experiments, the high-fat intake tended to lower plasma TG but not TC (Tables S3 and S4). As the fat content increased in the AFB diet with its low 0.35 P/S ratio, plasma TC and TG became elevated in the *susceptible*, but not in the *resistant*, rats. By contrast, increasing the amount of fat in the PKOB diet did not affect plasma lipids (Figure 2B and Figure 4B; Tables S5 and S6), in keeping with its P/S ratio of 1.0 and the favorable impact of a greater dietary P/S ratio on blood lipids. Because plasma lipids appeared more elevated in rats fed AFB, its low P/S ratio of only 0.35 presumably was a factor [45,46].

#### 3.1.8. Diet Effect on 18:2(n-6) %Energy and Gload

**Figure 5:**
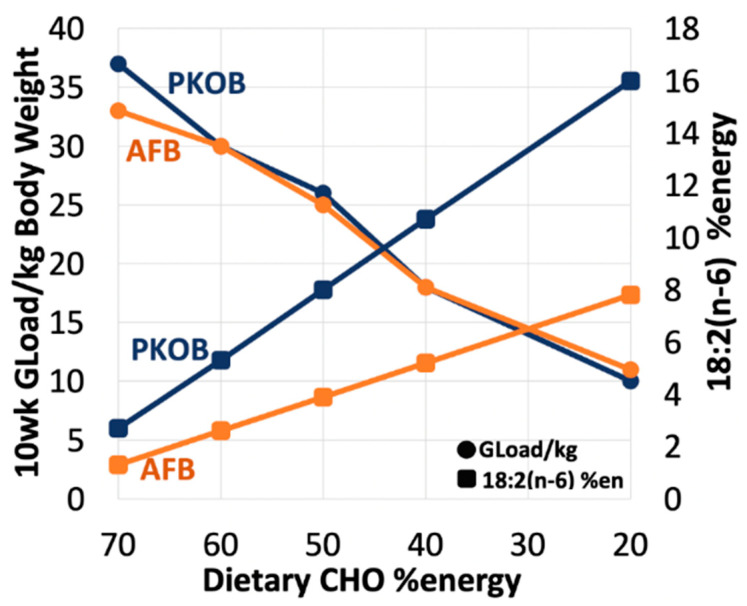
The GLoad (left Y-axis; dots) and dietary 18:2(n-6) intake (right-Y axis; squares) indicate the GLoad adjusted to BW decreased as CHO %energy decreased and the %energy as fat increased for all rats combined. The decrease in BW-adjusted GLoad was identical for both fats, PKOB (blue) and AFB (orange). By contrast, consumption of 18:2(n-6) was directly related to %energy from dietary fat, with the low CHO, high-fat diet providing maximum 18:2(n-6) at 16% energy for PKOB and 8% energy for AFB, both at 60% energy as dietary fat. Thus, the increase in 18:2(n-6) was twice as much for PKOB with a P/S ratio of 1.0, compared to AFB with a P/S ratio of 0.35, even though rats fed PKOB developed the most diabetes. Most (73%) diabetes for all rats occurred with diets providing 70, 60, and 50% energy as CHO, emphasizing the risk accompanying a high GLoad. Although PKOB induced 45% more diabetes overall than the AFB, the extra GLoad at 70% energy as CHO only partially explained the added risk for diabetes attributed to PKOB (see Tables S3 and S4 for details).

Another observation of interest was the relationship between 18:2(n-6) %energy intake and diabetes (Figure 5). Although aligned with total fat intake, 18:2(n-6) was inversely related to the above trends for diabetes. Thus, T2DM was most apparent at lower 18:2(n-6) intakes, which represented diets with hiCHO and less fat. By design, the PKOB diets provided more added polyunsaturated fatty acids (P/S of 1.0) than the AFB because the AFB was blended to mimic the current North American diet fat profile (P/S of 0.35). Thus, because PKOB provided twice the 18:2(n-6) content of the AFB, the greater diabetes associated with the PKOB diet would seem counterintuitive. Diets providing <8% energy as 18:2(n-6) signaled the breakpoint below which T2DM increased with the PKOB diets, whereas a threshold of <4% energy as 18:2(n-6) for AFB diets was associated with greater diabetes prevalence (Tables S3 and S4).

Not surprisingly, the GLoad and cumGLoad were greatest (the latter > 200/2000 kcal) for rats fed the 70:10:20 and 60:20:20 diets, but even >175 for rats fed 50:30:20. These measures of glucose intake decreased progressively across diets so that the GLoad was significantly lower (only one-half to one-third the amount) in the last two diet groups consuming the most fat and least CHO, i.e., 40:40:20 and 20:60:20. The GLoad further highlighted the importance of identifying *resistant* vs. *susceptible* rats in terms of diabetes outcome. For example, although both *resistant* and *susceptible* rats were offered the same GLoads when eating the same diet, the *resistant* rats, by definition and unlike their *susceptible* counterparts, did not respond with elevated RBG when consuming an excessive GLoad (>175/2000 kcal). This implies they exerted a certain genetic ‘resistance’ to T2DM. In addition, for any given diet, across all diet groups, rats were found to consume similar GLoads per day once adjusted for BW, with the exception of PKOB rats fed the greatest GLoad. This further suggests that body size and CHO intake should be evaluated in tandem in this model, at least during growth.

### 3.2. Analysis by Dietary Quintiles of Caloric Intake (Q_kcal_)

#### 3.2.1. CHO:Fat Score and Diabetes

The analysis based on diet composition indicated that caloric intake and BW gain/day were each affected by diet to impact diabetes outcome. Exploring the extremes of caloric intake by sorting rats into quintiles of average calories consumed per day (Q1–Q5_kcal_) revealed that genetic variation in body size at weaning was implicated in diabetes onset. This secondary analysis by caloric intake was inspired by previous observation [35,37] that greater consumption of daily calories was linked to increased diabetes. When rats in the current experiments were sorted by caloric intake (Q1–Q5_kcal_) in order to determine whether caloric intake or the initial analysis by diet composition was the better predictor of T2DM, the ranking revealed that neither the dietary CHO:fat ratio nor type of saturated fats (PKOB vs. AFB) predicted food intake. Instead, each quintile of energy intake (Q1–Q5_kcal_, *n* = 14 rats) reflected a mixture of 3, 4 or all 5 different dietary groups and their respective CHO:fat ratios. This unique mix of diets was used to reconfigure estimates of the newly combined CHO:fat ratios designated as the CHO:fat score for each quintile. In addition, a newly estimated CHO:fat:protein %energy was derived for those diets based on the 3–5 rats and their original diet assignment (Tables S7 and S8).

While the original analysis by diet composition produced dietary CHO:fat ratios that ranged from 7.0 (70:10) to 0.3 (20:60) across the 5 diets (Tables S3 and S4), the newly reconfigured ratios for the CHO:fat score per quintile (Q1–Q5_kcal_) produced a consolidated, narrower range between 3.5 and 1.0 (Tables S7 and S8). This energy sorted by quintiles revealed a positive relationship between calories consumed and diabetes induced (Tables S9 and S10). Furthermore, a hiCHO caloric intake by rats in a quintile also proved a consistent attribute underlying diabetes. For example, when rats fed PKOB consumed minimal calories (Q1_kcal_, avg 22 kcal/d), minimal diabetes occurred (15% diabetic rats), while diabetes in Q5_kcal_ was much greater (71% susceptibility, Tables S7 and S8). This was true despite the mix of diets assigned at weaning for the 14 rats in Q1_kcal_, which ironically happened to produce the highest estimated CHO:fat score (ratio of 3.4) and highest estimated CHO:fat %energy ratio (at 62:18:20) among all 5 quintiles in the PKOB experiment. However, Q1_kcal_ yielded the least diabetes because caloric intake for that quintile was so low. By comparison, Q5_kcal_ for PKOB had a CHO:fat score of 3.2 and an estimated CHO:fat ratio of 61:19:20, but induced the most diabetes due to the calories and GLoad consumed (Table S5). The point is that the dietary CHO:fat score or %energy ratio alone did not comprehensively explain diabetes induction. Caloric intake and GLoad needed to be considered as well.

#### 3.2.2. Diabetes Highlighted as RBG

**Figure 6:**
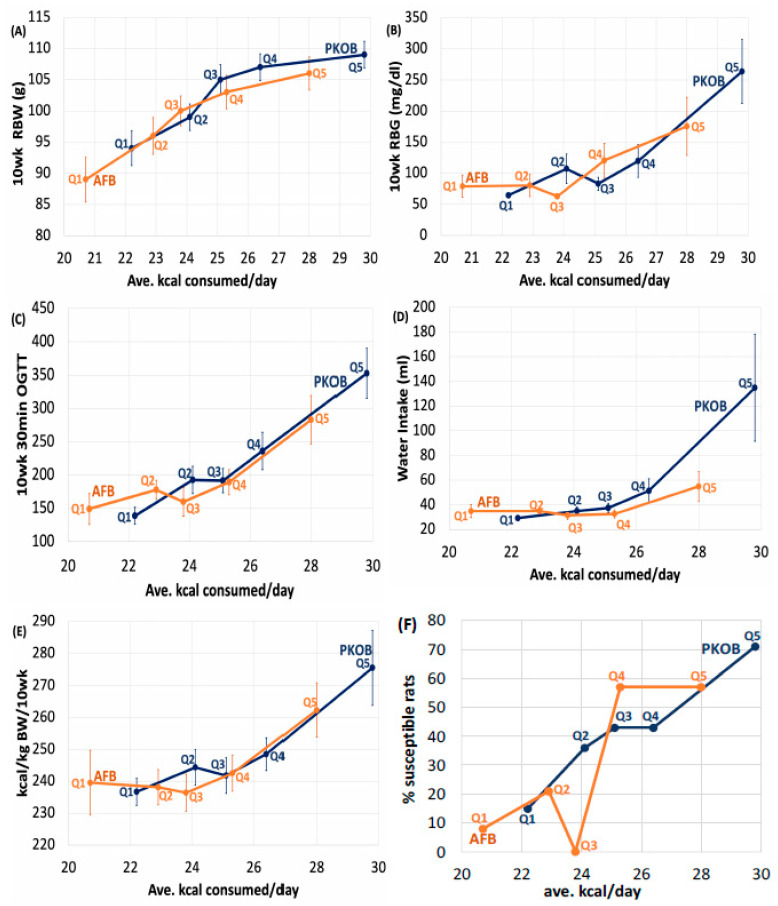
Clinical data for all rats fed either PKOB (blue) or AFB (orange) are expressed according to quintiles of caloric intake (kcal/d, *n* = 14 per quintile). (**A**) The average 10-week BW increased in a similar fashion for both fats across quintiles (Q1–Q5_kcal_, 20–30 kcal/d). (**B**) RBG began to signal diabetes (>75 mg/dL) when kcal intake exceeded 25 kcal/day, roughly beginning at Q3_kcal_ for both PKOB and AFB. (**C**) The 10-week 30′ OGTT indicated that diabetes began with Q2_kcal_ for PKOB and Q4_kcal_ for AFB at 25 kcal/day for both dietary fats. (**D**) Water intake was greater for rats fed PKOB than for rats fed AFB, and the increase occurred above Q4_kcal_ for both types of fat. (**E**) For all quintiles, the weight-adjusted caloric intake per quintile was at least 1 kcal/day greater for rats fed PKOB than those fed AFB. Weight-adjusted calories increased for rats above Q1_kcal_ for PKOB, but not until Q3–Q4_kcal_ for AFB. (**F**) The percentage of rats that were diabetic, i.e., *% susceptible*, rose rapidly with each quintile of caloric intake, above Q1_kcal_ for PKOB and above Q3_kcal_ for AFB. The three lowest quintiles of kcal intake for PKOB had appreciably more diabetes than the three lowest quintiles for AFB; however, the number of diabetic rats in Q4_kcal_ and Q5_kcal_ were similar for both fats, emphasizing the greater severity of diabetes in the three lowest Q1–3_kcal_ in rats fed PKOB (see Tables S7 and S8 for details).

In a trend similar to 10-week BW, diabetes (as RBG > 75 mg/dL) induced in rats in the highest caloric quintile (Q5_kcal_) for either PKOB or AFB was also more extensive than Q1–Q3_kcal_ (Tables S7 and S8). Interestingly, the range in RBG was much wider for the PKOB diet, with the lowest avg RBG of 64 mg/dL for Q1_kcal_ compared to an exceedingly elevated RBG of 264 mg/dL for Q5_kcal._ The AFB had a much narrower range, with an avg RBG of 82 mg/dL for Q1_kcal_ and 191 mg/dL for Q5_kcal_ (Figure 6B; Tables S7 and S8). This could imply a protective effect from AFB that prevented RBG from rising too high but also left the lower range of RBG more elevated. For the 30′ OGTT, rats in Q5_kcal_ that were fed either PKOB or AFB were significantly greater than those in Q1–Q4_kcal_ and Q1–Q3_kcal_, respectively (*p* < 0.05). Similar to the 10-week RBG, the range for 30′ OGTT was much less for AFB than PKOB (Figure 6B,C; Tables S7 and S8).

Thus, analysis by quintiles of caloric intake for rats fed PKOB revealed that the percentage of diabetic (*susceptible*) rats increased progressively from Q1_kcal_ to Q5_kcal_. For rats fed AFB, the trend was similar, increasing until Q4_kcal_, with Q5_kcal_ being about the same (Figure 6F). However, the PKOB diets with more 8C–12C fatty acids, i.e., MCTs, Q1–3_kcal,_ generated 3 times as many diabetic rats (13/41, or 32%) compared to AFB with only 4/41, or 10% (Tables S7 and S8). This observation suggests that 8C–12C behaved like MCTs and glucose and were transported via the portal vein directly to the liver for immediate processing. Another possibility is that the composition of long-chain fatty acids present in the animal-based AFB may have reduced the risk from hiCHO at normal caloric intakes (21–24 kcal/day) by generating specific phospholipid or ceramide structures that provide better protection against insulin resistance and hiCHO-induced diabetes [30].

#### 3.2.3. Caloric Quintiles (Q_kcal_) Sorted as Resistant/Susceptible Rats

Even though our analysis by diet composition that focused on the CHO:fat ratio revealed diabetes was reduced when high-fat displaced refined CHO, diabetes in individual rats appeared to be affected by epigenetics when co-existing with caloric consumption expressed by the Q1–Q5_kcal_ distribution. A previous study [35] revealed a propensity for the most *susceptible* rats to eat the most calories, and this relationship was evident from the start of the current experiment once food intake and growth rate were evident within one to three weeks post-weaning. Thus, a genetic influence over caloric intake appeared important from the beginning, before the experimental diet assignment could be expected to exert an impact.

By virtue of the quintile sort for energy intake (Q1–Q5_kcal_), *resistant* and *susceptible* rats that were distributed into the same quintile ate the same number of calories, or approximately 21–24 kcal for rats in Q1 and Q2_kcal_ and 25–30 kcal for rats in Q3–Q5_kcal_ (Tables S9 and S10). This re-evaluation of susceptibility in terms of energy intake was telling because rats in Q4–Q5_kcal_, where *susceptible* rats were most abundant and represented more than 60% of all diabetic rats, also revealed that the *susceptible* rats happened to be randomly assigned at weaning to diets with greater-CHO and less fat than the *resistant* rats in those same quintiles of caloric intake (Tables S9 and S10).

The diet code also revealed that 11–12 *resistant* rats in Q1_kcal_ for both experiments were represented by 3 of the 5 possible PKOB and AFB diets, mostly 70:10 and 40:40, presenting a relatively high CHO:fat score of 3.2 and 3.5, respectively. Q5_kcal_ generated a greater CHO:fat score of 3.5 for *susceptible* rats fed PKOB, compared to a score of 2.3 for *restsistant* rats fed PKOB. Out of the available diets, the 70:10, 60:20 and 50:50 hiCHO diets represented all the *susceptible* rats fed PKOB. By contrast, the *resistant* rats consumed diets with a wide range of fat and CHO (Tables S9 and S10). For *resistant* rats fed AFB, the CHO:fat score declined sharply by chance from 3.5 in Q1_kcal_ to 1.0 in Q5_kcal_ due to their diet assignment, as seen in the diet code. On the other hand, *susceptible* rats across AFB diets averaged a CHO:fat score of 2.5, again as a result of their random assignment to diets, which was reflected in a greater number of rats in the Q5_kcal_-*susceptible* subset that consumed hiCHO diets, or stated another way, by chance *resistant* rats with high caloric intakes that had been randomly provided low-CHO, high-fat diets at weaning, in effect subjecting them to more fat calories and less CHO stress during the experiment (Tables S9 and S10).

*Susceptible* rats (Q3–5_kcal_) also expressed their phenotypic propensity for T2DM early on by growing faster and eating more food than rats that ended up in Q1_kcal_ and Q2_kcal_. The greater caloric intake by rats in Q3–Q5_kcal_ was reflected in the final BW and diabetes, i.e., heavier *susceptible* rats expressed the most severe diabetes. As a consequence, the genetics/epigenetics underlying susceptibility appeared to play a major role by controlling food intake and growth rate that was reflected in glucose dysregulation and diabetes (elevated RBG, OGTT) if diet assignment favoured hiCHO intake. By the same token, *resistant* rats could eat robustly and grow rapidly yet not develop diabetes if a diet with high GLoad had not been randomly assigned at weaning as an added dietary risk factor. Alternatively, due to genetics affecting appetite control, *resistant* rats could eat sparingly and thereby avoid the risk of dietary CHO overload. It is also possible that resistance might reflect undisclosed genes, i.e., related to *genetic permissiveness*, that could better cope with a dietary glucose overload once absorbed. The greater BW that tended to characterize *susceptible* rats fed either type of fat was usually apparent within 3 weeks on the challenge diet (6 weeks of age) for most diets [1,35,38,39].

Interestingly, when sorted by quintiles of average kcal consumed/day, *resistant* rats on average consumed about 22–25 kcal/day across all 5 diet groups in both studies, while *susceptible* rats tended to average 10–20% more, i.e., 26–29 kcal/day, except for *susceptible* rats fed the high-fat PKOB diet (60:20:20 CHO:fat:protein %energy) that tended to eat less than *resistant* rats in that diet group (Tables S9 and S10), perhaps losing appetite as ketosis developed in advancing diabetes, signaled by weight gain reduction between 6 and 10 weeks on the diet compared to AFB rats. This further demonstrates the strong *genetic permissiveness* coupled with diet fat type at work here.

The point is that diabetes was effectively avoided by individual *resistant* rats that exercised central nervous system (CNS) control over energy intake and subsequently ended up in the slow-growth, *resistant* category, as opposed to food intake being regulated based on a specific diet composition reflected in the CHO:fat ratio. No evidence for the latter argument was observed.

Furthermore, sorting rats as *resistant* or *susceptible* within caloric quintiles revealed that the %*susceptible* rats increased progressively across each Q_kcal_, ranging from 8–15% in Q1_kcal_ to 57–71% in Q4–Q5_kcal_ for the AFB vs. PKOB diets, respectively. *Severity* was more individualized but also tended to be greatest for rats in Q4–Q5_kcal_, i.e., where the caloric intake was greatest. Another characteristic of *severity* was that all *resistant* rats improved their RBG and OGTT between 6 weeks and 10 weeks of the study, while the *susceptible* rats became progressively more diabetic as they matured (Tables S7–S10). This aspect of susceptibility was observed previously in young, growing Nile rats [35], which suggests that enzymes and hormones involved with glucose absorption and metabolism improved with age only in the *resistant* rats, even as *susceptible* rats became increasingly overwhelmed and more glucose impaired. Accordingly, hiCHO intake associated with any diet, especially those providing 70%, 60% and 50% CHO energy, increased the odds of developing diabetes in *susceptible* rats if those rats, for reasons of *genetic permissiveness*, also consumed more calories (Q4–Q5_kcal_), but not so much if a rat ended up in the lower quintiles of caloric intake (Q1–Q3_kcal_), even if it happened to receive a hiCHO diet. In the latter case, the outcome was 4-fold worse if that diet fat happened to be PKOB. This point places further emphasis on the importance of caloric intake as an important consideration in diabetes outcomes.

In essence, *susceptible* rats that ate the most weight-adjusted calories, i.e., in Q5_kcal_, were revealed by this secondary analysis based on caloric intake as those eating a composite of diets that generated the highest CHO:fat score and diet code in their respective quintile. In effect, they had been assigned randomly as weanlings to diets with the greatest GI (Tables S9 and S10). Their genetics allowed them to grow faster and eat more such that caloric intake exacerbated their diabetes risk if the diet assigned happened to be CHO-rich. The clearest divergence in this weight-adjusted risk was revealed by comparing *resistant* and *susceptible* rats across quintiles of caloric intake between Q1_kcal_ to Q5_kcal_, but especially noticeable in Q4 and Q5_kcal,_ where caloric stress was most concentrated (Tables S9 and S10). The point is that only by sorting rats both by caloric intake and then by their genetic propensity to consume those extra calories dictated by growth, in essence by separating *susceptible* from *resistant* rats across Q1–Q5_kcal_, was it possible to comprehend the complex interrelationship that depended on the inherent trait modulating growth and food intake. In the end, genetics/epigenetics, i.e., *genetic permissiveness*, proved crucial, but dietary CHO as GLoad was required to demonstrate it.

#### 3.2.4. Kcal Sort on BW and Food Efficiency

Sorting BW by quintiles of kcal/day (Q1–Q5_kcal_) also rendered the association between BW (growth rate) and diabetes more apparent, including a subtle effect of fat type. In general, rats in each caloric quintile for the PKOB diet were heavier and more diabetic than the corresponding caloric quintile for rats fed the AFB diet. Not surprisingly, rats that consumed the most kcal/day (Q4_kcal_ and Q5_kcal_) revealed the greatest daily BW gain. This association between weight gain and T2DM induction was noted previously [1,35,38,39]. The weight gain across quintiles in our data was mostly due to adipose weight, although the effect was modest. Liver weight increased, but not more than the adipose.

Furthermore, food efficiency, which reflects the metabolism of energy required to gain a gram of weight during the period of rapid growth (1–6 weeks), did not appear to affect rat classification as *resistant* or *susceptible* (Tables S9 and S10). Thus, each rat ate and grew (BW and BMI) in a predictable, physiologically normal fashion once total calories were accounted for and adjusted for BW.

#### 3.2.5. Kcal/Day Relates to Dietary cumGLoad

Sorting the incidence of diabetes based on the 10-week RBG <75 mg/dL>, i.e., into *resistant* and *susceptible* rats, further revealed hiCHO diet and GLoad as key components of diabetes risk. Although the cumGLoad for rats in Q5_kcal_ fed PKOB was significantly greater than that for rats in Q1–Q4_kcal_, this was not true for rats fed AFB diets (Tables S7 and S8). This might imply that the plant-based saturated fatty acids rich in 8C–12C behaved more like carbohydrates with hepatic portal vein transport directly to the liver during absorption, conferring more deleterious effects on hepatic metabolism and resulting in 4x the rate of T2DM in rats fed with hiCHO (70–60–50 %energy) PKOB diets compared to rats fed those 3 diets containing the AFB. It is also of note that Q1_kcal_ rats fed either PKOB or AFB ate a mix of hiCHO diets similar to Q5_kcal_ rats yet failed to develop much diabetes because the amount of food consumed, including the cumGLoad, was too meager in Q1_kcal_ and did not stress CHO metabolism. By contrast, the greater GLoad consumed by rats in Q5_kcal_ induced the most diabetes for both fat types. This further emphasizes that rats prone to eating the most calories were at added risk if also assigned to a hiCHO diet when weaned, exposing the worst-case, high-risk combination of hiCHO intake and extra calories.

It is also of note that the total calories consumed/day by corresponding quintiles for PKOB and AFB were similar, ranging from about 21 kcal/d for Q1_kcal_ to almost 30 kcal/d for Q5_kcal_, which implies other comparisons between the two fat studies can be considered robust, as well (Table S7). The hyperphagia in Q4_kcal_ and Q5_kcal_ proved to exert substantial diabetes risk, which was similar for both fat types at Q4–Q5_kcal_, but especially if the extra calories from the randomly assigned diet at weaning substantially increased the cumGLoad (Tables S7 and S8). The growth response associated with quintiles of caloric intake (Tables S7 and S8) was also linked to available dietary glucose, which was ultimately reflected in the cumGLoad.

However, like calories, neither the GLoad nor cumGLoad alone provided a complete explanation for diabetes risk when analysed only in terms of diet composition because diet by itself failed to account for the calories consumed. The GLoad typically exposed diabetes risk only when caloric consumption averaged >25 kcal/day, and the assigned diet composition resulted in a cumGLoad that exceeded the glucose limit for a genetically-controlled diabetic threshold in that rat, i.e., in Q3_kcal_ to Q5_kcal_, which was reflected in the RBG and 30′ OGTT. In summary, risk exposure was exacerbated if dietary CHO averaged more than 57% energy, fat averaged less than 25% energy, and the cumGLoad exceeded 175/2000 kcal of diet.

From the dietary point of view, a rat became diabetic, or not, depending on the hiCHO content of the calories coupled with a propensity to consume the most calories, further emphasizing the combination of hiCHO and robust consumption as the problem. In fact, daily caloric intake was just as important as the diet sourcing those calories, and the amount consumed seemed to be driven by genetics/epigenetics and growth demands. Furthermore, excess calories as CHO were much more damaging than fat calories in terms of T2DM risk expressed as RBG and 30′ OGTT, as well as organ weights at necropsy.

#### 3.2.6. Kcal Effect on Water Intake

Water intake revealed a similar result, where rats that ate the most calories gained the most weight, expressed the most diabetes (Q5_kcal_), and drank significantly more than Q1–Q4_kcal_ for both PKOB and AFB diets. However, much like BW data, the range of water consumption for PKOB (29–135 mg/dL) was greater than the range for AFB (30–60 mg/dL). Water intake for Q5_kcal_ rats fed PKOB was significantly greater than water intake for Q5_kcal_ rats fed AFB. This again implies a more deleterious effect of PKOB on T2DM and kidney function when PKOB was consumed with excess calories as hiCHO diets (Figure 6D).

#### 3.2.7. Kcal Effect on Organ Weights

**Figure 7:**
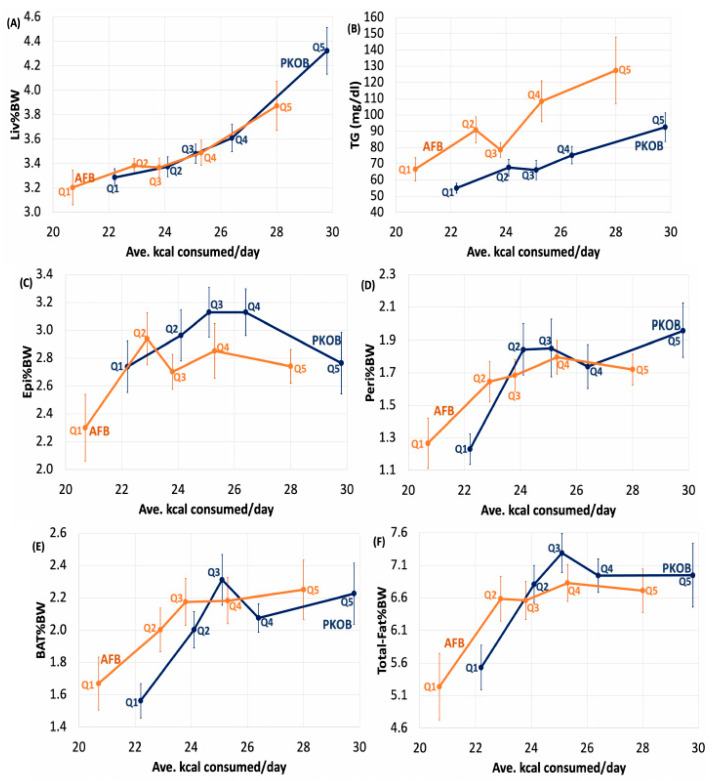
Necropsy and plasma TG data for rats fed either PKOB (blue) or AFB (orange) are ranked according to quintiles of kcal/d on the X-axis. (**A**) Liver as %BW reveals a steady increase for both fats as kcal/day intake increased, more pronounced for PKOB than AFB. Liver weight tracked BW as depicted in Figure 6A. (**B**) Terminal plasma TG, although not severe, revealed greater triglyceridemia for AFB than PKOB at all Q_kcal_, reflecting their P/S ratios of 0.35 and 1.0 for the two fats, respectively. (**C**) Rats fed PKOB accumulated more epididymal fat than AFB rats at all quintiles, except quintile Q2_kcal_. (**D**) Perirenal fat was greater for PKOB than AFB, again reflecting caloric intake. (**E**) At lower caloric intakes, i.e., less than 25 kcal/d where minimal diabetes occurred for AFB, BAT tended to be more abundant than for PKOB rats, but BAT reserves plateaued and were similar between fats starting at 25 kcal/d once diabetes increased for both fats. (**F**) Total body fat pools tended to be similar for matching Q_kcals_ for both PKOB and AFB, but PKOB rats ate about 1 kcal/d more than AFB per quintile and thus had more body fat overall, in concert with more diabetes at greater caloric intakes (see Tables S7 and S8 for details).

Even though greater consumption of calories led to fatty liver, in general, and PKOB (8C–12C fatty acids as MCT) clearly produced more fatty liver than AFB, organ weights and adipose tissues revealed that Liv%BW was significantly greater in Q5_kcal_ compared to Q1–4_kcal_ for both PKOB and AFB. However, the range of Liv%BW was less for rats fed AFB (3.1–4.0) compared to PKOB (3.3–4.3) (Figure 7A). In addition, for Peri%BW, BAT%BW, and total fat %BW, the lowest caloric intake (Q1_kcal_) generated significantly lower values than Q2–Q5_kcal_, regardless of the type of fat. Thus, caloric intake itself, independent of dietary fat type, was definitely related to T2DM risk and was reflected in fat accumulation.

#### 3.2.8. Kcal Effect on Plasma Lipids

Plasma TG revealed that even though, on average, the PKOB rats consumed more kcal/day than AFB rats, the latter consistently had higher plasma TG for each quintile. This implies that whereas PKOB presented more risk for diabetes and Liv%BW (fatty liver), the AFB appeared more deleterious in terms of the blood lipid profile, including the current re-emphasis on cardiovascular risk from hypertriglyceridemia [57].

### 3.3. Analysis by Quintiles of RBG <75 mg/dL> across Diets

#### 3.3.1. RBG (Q1–Q5_RBG_)

While the analyses by diet composition and caloric intake emphasized the importance of calories over diet composition, both identified the underlying influence of genetic permissiveness that likely biased diabetes outcome via the expression of genes that, in part, dictated caloric intake and subsequent glucose disposal following food consumption. To explore these relationships more fully, all rats in each experiment were sorted into quintiles of RBG (Q1–Q5_RBG_) at the end of the study to examine whether an inherent genetic or epigenetic trait might be better exposed, i.e., would diabetes rank (as RBG) accentuate diet composition or response traits such as growth rate or calorie intake linked to diabetes and NAFLD. In a previous study, RBG ranked by quintiles proved insightful [35]. Thus, data were pooled in each of the two current studies and evaluated across quintiles of RBG from low (Q1_RBG_) to high (Q5_RBG_).

#### 3.3.2. RBG Quintiles (Q1–Q5_RBG_) Separates Resistant/Susceptible Rats

When sorted into quintiles of pooled rats by RBG (Q1–Q5_RBG_), the range was wide, from 51 mg/dL in Q1_RBG_ to 353 mg/dL in Q5_RBG_ for all 138 rats covering both experiments. Because most rats in Q1–Q3_RBG_ had RBG values < 75 mg/dL, those 3 quintiles by definition were designated as *resistant* to T2DM, while rats in Q4&Q5_RBG_ were considered *susceptible*, with Q5_RBG_ at 269 mg/dL and 353 mg/dL for AFB and PKOB, respectively (Tables S11 and S12).

#### 3.3.3. CHO:Fat Score, Gload, BW and 18:2(n-6)

Sorting RBG by quintiles again generated a CHO:fat score and diet code for each quintile, providing another way to assess the diet contribution to diabetes. For the PKOB diet, rats in Q2_RBG_ generated the lowest CHO:fat score at 1.6 ± 1.8, while Q3_RBG_ revealed the greatest score at 3.1 ± 2.7. Scores were estimated from their projected diet CHO:fat:protein ratios generated from the 5 different diets consumed by the 14 rats/quintile. These ratios for Q1_RBG_ and Q3_RBG_ were calculated to be 49:31:20 vs. 60:20:20, respectively, in turn representing GLoads of 163 vs. 208/2000 kcal of diet. The point is that neither the CHO:fat score nor the GLoad alone, based on pooled diets sorted as quintiles of RBG, were effective predictors of the aveRBG (diabetes induced) per quintile (Tables S11 and S12).

In essence, none of the parameters, such as growth, food intake, etc., disclosed by the sorted RBG quintiles, were clear predictors of diabetes outcome after adjusting for kg BW, with the weak exception of the GLoad per day for Q5_RBG_. Thus, although BW tended to reflect the final RBG for Q1–Q5_RBG_, caloric intake (and GLoad) tended to be increased only for rats in Q5_RBG_, but even that relationship lost significance after the BW adjustment. In other words, the largest rats ate the most calories and grew most rapidly because they were larger as weanlings (genetics), not in response to the diet composition consumed during the test period. Additionally, like the previous analysis based on diet, no strong relationship was found between 18:2(n-6) intake and RBG rank, although it tended to be slightly inverse, with a greater 18:2(n-6) intake associated with a lower 10-week RBG.

#### 3.3.4. Organ Weights and Plasma Lipids

Liver weight correlated with RBG elevation, and the largest livers were associated with rats in Q5_RBG_, i.e., the most diabetic quintile. Additionally, body fat was 15–20% greater in Q4 and Q5_RBG_ for both fat types, but BMI was the same across Q1–Q5_RBG_. When slightly increased, the BMI reflected organ size, not body fat. Thus, obesity did not appear to be a factor in either study for either type of fat. Finally, the plasma lipids, as TC and TG, were elevated for Q5_RBG_, indicating that MetS and fatty liver (NAFLD) were associated with diabetes once again, this time defined by quintiles of RBG (Tables S11 and S12).

In summary, sorting the RBG into quintiles from low (Q1_RBG_) to high (Q5_RBG_) revealed only Q5_RBG_ as seriously elevated and predictive of clinical diabetes for most of the parameters assessed, including water intake. As such, the ranking by RBG furthered the notion that caloric intake driven by body size and *genetic permissiveness* were critical factors in predicting diabetes outcomes.

## 4. Discussion

Growth is proving to be a key factor for Nile rat diabetes expression because it features food intake in a dynamic phase of the test period when genetic control over energy intake (appetite, hormones) is most crucial and presumably in a state of flux, i.e., much more than the static adult maintenance energy requirements would ever influence food intake acutely. If food consumption is regulated adequately by appetite control during growth, T2DM induction can be effectively avoided. This must be true at any age but is less readily detected in adults, e.g., in adult humans where most such nutritional studies have been conducted.

### 4.1. CHO and GLoad

In a previous report, when 3-week-old male Nile rats were all fed the same semi-purified hiCHO diabetogenic diet (60:20:20), and the diabetes outcome was ranked as quintiles of RBG, rats with the most diabetes also consumed the most calories during the 10-week diet challenge [35]. Thus, Nile rats in Q5_kcal_ of that study, with average RBG > 425 mg/dL, ate more calories and proved to be more genetically prone to diabetes than rats in Q1_kcal_ (RBG 52 mg/dL), which ate the fewest calories. The trend appeared linear from the 1st to 5th quintile, with notable increases in both average kcal/day and diabetes (RBG) observed in the 4th and 5th quintiles after 10 weeks. Because of the nature of the hiCHO diet in that study, the original assumption was that diets with a high GI, and a dietary GLoad > 175 per 2000 kcal, would induce diabetes in the absence of dietary fiber. However, the single-diet design and common CHO:fat ratio fed to all rats (3.0, for a 60:20:20 diet) in that original study [35] failed to determine whether increased T2DM, and its associated kcal/day intake, were due to the lack of fiber, the hiCHO diet, or a low dietary fat content possibly limited in EFAs. Thus, it was not possible to establish which macronutrient factor was the underlying reason for excess food consumption and diabetes or even whether a macronutrient component was definitively linked to diabetes outcome.

Therefore, to explore the CHO:fat issue further, the present study design tested whether progressive fat substitution for CHO might partially explain the difference in diabetes incidence. The high-fat, low-CHO diets, as 40:40:20 or 20:60:20, did tend to reduce diabetes susceptibility, but the answer proved more complicated because although the caloric intake was important, two additional covariates needed to apply:

(1) The extra calories had to reflect an increased GLoad within an overall increase in caloric intake, and
(2) Individual host genetics/epigenetics had to be vulnerable to processing the increased glucose burden.

In other words, a metabolic system overloaded with glucose resulted in MetS and T2DM, especially when analyzed as quintiles of kcal consumed/day or quintiles of RBG. Nile rats that grew more quickly and consumed more kcal/day due to presumed genetic/epigenetic demands affecting growth and appetite were more apt to develop T2DM if those extra calories resulted in more than 55% energy as processed CHO, e.g., 60:20:20 having a CHO:fat score of 3.0. When presented with a diet with a CHO:fat score > 2.7, these rats were considered *genetically permissive* if they were unable to metabolize the excess glucose burden effectively. In essence, dietary CHO was needed for diabetes to occur, and a GLoad greater than 175–200 per 2000 kcal seemed critical to inducing diabetes in genetically prone Nile rats.

### 4.2. Dietary Fat

Viewing the present experiments from the dietary fat point of view, it was surprising that neither the amount nor type of fat, including fatty acid unsaturation per se, appeared to exert much effect on T2DM within a normal range of fat intake (approximately 20–40% energy as fat). Furthermore, increasing total dietary fat as either PKOB or AFB at the expense of CHO did not lead to overconsumption of calories and/or fatty liver. Hence, fat calories were not particularly important unless fat content was exceptionally high at 60% energy, when it displaced CHO to <40% energy to indirectly result in less diabetes among *susceptible* rats.

The displacement of CHO with excess fat (20:60:20) caused the cumGLoad to be <85/2000 kcal of diet, which, in turn, deterred diabetes and reinforced the concept that T2DM does not develop if CHO is sufficiently displaced by dietary fat. That observation is in keeping with ketogenic diets that emphasize the anti-diabetic aspect of fat [17,58,59,60,61]. In effect, fat had minimal impact on T2DM in this Nile rat study. This conclusion was supported from different perspectives in both experiments, where results were initially based on the five diet compositions to determine whether greater fat and less CHO would reduce the incidence (%*susceptible*) and *severity* of T2DM. Because saturated fat has consistently been linked to T2DM in the past [32,34,62], two types of saturated fat providing two distinct levels of polyunsaturated fatty acids were compared for their impact on overall diabetes *susceptibility* and *severity*. PKOB tested diabetes induction with a plant-based saturated fat rich in 8C–12C fatty acids plus added soybean oil to balance the P/S ratio at 1.0 [46], whereas AFB represented a blend of animal saturated fat rich in 16C–18C fatty acids balanced with soybean oil to generate a fat blend with a P/S ratio of only 0.35 similar to the US average, considered more stressful on lipid metabolism. The P/S ratio of the latter fat was intentionally one-third that in PKOB, assuming that the historical disadvantage of a low P/S ratio < 0.4 on lipoprotein metabolism might pertain to T2DM as well [45,46]. It did not.

In fact, the overall *%susceptibility* for diabetes was 45% greater with the PKOB than with AFB (42% vs. 29% incidence), even though the plasma lipids tended to be greater following consumption of the saturated animal fat blend. The latter observation confirmed the well-known deleterious effect of a lower P/S ratio on lipemia in animals and humans [45,46].

The implication is that the type of dietary saturated fatty acids may affect glucose and lipid metabolism via unique metabolic pathways while further emphasizing that the overall composition of fat and the P/S ratio, in general, had minimal effect on T2DM induction. Nevertheless, another disconnect was that the lowest intakes of 18:2(n-6) %energy in experiment 1 and experiment 2 tended to associate with more T2DM. However, for both experiments, the lowest intake of 18:2(n-6) was well above a level that might elicit EFA deficiency, i.e., at 2.7% energy as EFA, and although PKOB had twice the 18:2(n-6) %energy as AFB for each of the 5 diets, the PKOB diet induced more diabetes, so 18:2(n-6) intake did not appear important for diabetes outcome if adequate EFA were consumed.

Because PKOB seemed to be more diabetogenic than the AFB, it is possible that TGs rich in 8:0, 10:0, and 12:0 in PKOB acted more like CHO by favoring the direct transport of these medium-chain triglyceride (MCT) fatty acids to the liver via the portal vein as opposed to lymphatic absorption via chylomicrons used by long-chain fatty acids in the AFB [63,64]. This implies that hepatic phospholipids or ceramides made with MCTs rich in 8C–12C fatty acids are not as beneficial as those derived from 16:0 + 18:0 when it comes to glucose metabolism [27,29]. In that sense, intake of PKOB resulting in greater liver fat content may have an increased burden on hepatic glucose metabolism, especially at higher CHO intakes.

### 4.3. Minor Components

These Nile rat studies were designed to have no confounding by fiber or phytonutrients, and both were excluded as supplements from all diets. Protein was constant in both studies at 20% energy and was provided by high-quality milk proteins. Fat quality proved to be minimally important in our design, possibly because balancing dietary fats with soybean oil prevented any complications from EFA deficiency, even though fat composition was sufficiently diverse to affect blood lipids negatively, e.g., saturated fat as AFB raised TC and TG in *susceptible* rats. An inverse relationship between the GLoad and 18:2(n-6) intake was exposed in *susceptible* rats, as well. Thus, our designs clearly separated diabetes risk attributed to CHO and the cumGLoad, while providing minimal risk attributed to fat in the comparison of the two key macronutrients. In effect, excess CHO, not fat, was implicated as the main dietary factor influencing the incidence of T2DM and its *severity*.

### 4.4. Caloric Intake Issue

Further analysis beyond the dietary CHO:fat ratio revealed that T2DM induction occurred primarily in Nile rats that consumed the most calories (ave > 25 kcal/day) coupled with a GLoad sufficiently great to trigger an overload of CHO calories in terms of the cumGLoad per rat, most notably in diets containing PKOB. When initially analyzed on the basis of diet composition alone for *all* rats, the impact of GLoad on T2DM disappeared once corrected for BW, an observation that was attributed to the large variation in RBG and calories between *resistant* and *susceptible* rats that occurred among individual rats for any given dietary CHO:fat ratio. It required a more robust assessment of calorie intake into quintiles to establish that rats consuming a normal amount of calories, i.e., Q1–Q2_kcal_ at 22–24 kcal/day for 10 weeks, ended up *resistant* to T2DM, whereas those consuming >26–30 kcal/d as in Q3–Q5_kcal_, were prone to diabetes independent of a specific diet macronutrient composition, but only if the individual rat consumed a diet where the GLoad > 175/2000 kcal. Sometimes rats fed high-fat diets ate more than 25 kcal/day, even falling into Q4_kcal_ or Q5_kcal_ for caloric distribution, yet they still avoided diabetes because the CHO content of those calories was low, i.e., diets with 40:40:20 or 20:60:20, and hence below the necessary GLoad threshold of 175/2000 calories. This observation further emphasizes the qualifying interrelationships that existed between diabetes induction and total caloric intake, dietary GLoad, cumGLoad per individual rat, and the dietary CHO:fat ratio.

The point is if an animal in any species, including humans, consumes minimal daily calories as CHO, diet-induced diabetes will not normally develop because the liver cannot generate enough glucose from fat and protein to induce hyperglycemia. This explains the rationale for the low-CHO, high-fat ketogenic-diet concept [17,19,65,66,67,68]. However, such diets are not without risk, as chronic high-fat diets reportedly can damage the gut mucosa and microbiome to induce ‘leaky gut’ syndrome [69]. An exception to the norm, of course, which has resulted in great confusion about diet-induced diabetes, are animal models such as mice and conventional rats that require high-fat, moderate-carb diets for T2DM induction [1].

In those instances, such rodents overconsume fat calories and lose control over their caloric intake to become obese, leading to insulin resistance and diabetes. The modest GLoad in those diets ultimately causes diabetes secondary to obesity and insulin resistance. However, it is important to remember that only about 50% of humans with obesity and insulin resistance ever become clinically diabetic after losing glycemic control, presumably because their GLoad never fully compromises their individual genetic ability to produce enough insulin, however minimal, and thereby they are able to metabolize the glucose burden consumed during daily fasting intervals [70,71,72].

To the above point, dietary fat and protein alone will not induce T2DM [17,58,59,66,67,68,72,73,74]. A second point is that an individual does not have to consume a ketogenic diet to prevent diabetes if the usual diet and brain satiety center limit caloric intake sufficiently to allow the dietary glucose load to be metabolized adequately per unit time [72]. Accordingly, a major implication from these experiments on T2DM induction is that diet composition related to the CHO:fat ratio in our fiber-free diets appeared not to be as critical as the mass of food (calories) consumed; secondly, the mass of CHO in that caloric burden had to exceed a certain percentage of total energy metabolized (typically > 57% energy as refined CHO) to cause an excessive GLoad to induce diabetes in the genetically susceptible host. Thus, neither the GI nor the GLoad alone, nor a specific diet composition per se, predicted T2DM outcome so much as the underlying genetic control over food intake that dictated the total energy and, ultimately, the cumGLoad metabolized daily [75], although GLoad was a cofactor that favored overconsumption [75].

Whether hiCHO diets as ultra-processed foods actually cause overeating to occur needs further investigation. Furthermore, diabetes induction depended somewhat on intake of extra calories based on body size, reflecting the individual rat’s ability to control food intake, linked to the genetic, hypothalamic control over caloric intake in these weanling Nile rats during growth. This apparent genetic or epigenetic control was observed as early as 2–3 weeks post-weaning [35] and actually seemed to improve with age in *resistant* rats, but not in *susceptible* rats, where control over caloric intake and satiation continued to deteriorate.

Thus, the rats that ate the most food had a greater chance to consume more CHO and cumGLoad and increase their risk for T2DM when their assigned diet was sufficiently rich in CHO at CHO:fat ratios of 70:10, 60:20 and 50:30. This overwhelmed glucose metabolism and led to MetS. An evaluation of dietary fiber is missing here, but previous Nile rat data demonstrate that high fiber intake can decrease the GI and GLoad and completely block T2DM induction despite a hiCHO diet (70:10:20) [39], which is in keeping with the current consensus about the importance of fiber in human diets [76].

A key point is that the actual signal for control over food intake in the current studies did not seem related to diet macronutrient composition or quality, including the fat P/S ratio or the dietary 18:2(n-6) %energy intake [45], when dietary fiber and phytonutrients were avoided by design. Instead, average kcal/day intake appeared to be influenced by the inherent growth rate and related, but unmeasured, gut-brain signals controlling appetite, which are likely interconnected by cross-talk between select tissues, gut microbiome, and brain [19,72,73,74,75]. Furthermore, the reformulated hypothetical CHO:fat score and the estimated CHO:fat:protein %energy extrapolated after reassigning rats to quintiles of caloric intake were similar for caloric quintiles Q1_kcal_ and Q5_kcal_ in experiment 1, even though the calories eaten and diabetes induced differed the most between those two quintiles based on what appeared to be epigenetic factors controlling caloric intake. The specific genes affecting the outcomes were not assessed nor disclosed by the current study design. Simply stated, diet composition beyond its related cumGLoad was a weak predictor of T2DM relative to the purported epigenetic factors impacting energy intake, presumably involving the microbiome, based on the observed differences in cecum weight. This underscores the importance of the GLoad presented by the typical diet of a given population [12,76] but does not address the more relevant relationship between diet composition or texture and regulation of food intake by individuals, assuming there is one.

### 4.5. Human Data Support

Concerning humans, one would like to know what attributes of diet contribute to the dysregulation of food intake in humans that parallel the Nile rat findings. An underlying problem likely rests with ultra-processed foods associated with Western diets that have low P/S ratios and disrupt or remove plant-based fibers and antioxidants as phytonutrients, components that substantially benefit the microbiome [12,76,77] and, in turn, contribute to the regulation of whole-body energy metabolism and food intake [78,79]. Such distortion in energy metabolism in humans is supported by the observation that highly-processed foods can result in excessive caloric intake. More specifically, it is an overload of highly-processed CHO-rich calories that proves deleterious [7,62,80,81,82].

#### Epidemiological Data

The conclusion that excessive caloric intake in Nile rats results in weight gain and metabolic syndrome leading to T2DM has precedent in recent human epidemiological data [62]. For example, diet and macronutrient composition for more than 10,000 middle-aged Chinese subjects was assessed with basically no relationship observed overall between T2DM and diet macronutrient composition despite an overall hiCHO content at 61:27:12 for CHO:fat:protein %energy. However, further in-depth analysis found that *susceptible* subjects ate the most calories (25% more) but developed diabetes at even lower CHO intakes [52:36:12] than the general population. In addition, their diet was enriched in animal products, including protein quality, and was lower in plant-based fiber and phytonutrients. The latter is in keeping with the present Nile rat data.

Unlike the epidemiological studies from China [62] and the US [42,83], the diet protein in the present Nile rat study was held constant at 20% energy and had a consistent quality to milk proteins. Thus, unlike the human epidemiological studies, protein was not a complicating variable in the Nile rat study design focused on CHO and fat.

Because our rat study did not include calories derived from meat, the implication from the Chinese study that meat consumption itself was a risk factor was not tested in Nile rats, leaving high caloric intake and low plant-based foods as the key common variables. In the Chinese study, too many variables, i.e., plant vs. animal fat and protein, and CHO GLoad, including fiber and phytonutrient intakes, were assessed simultaneously by virtue of the observational nature of the study, making conclusions about specific nutrients, including the GLoad, impossible [62].

However, with more control over macronutrients, the Nile rat data suggest that diabetes in the Chinese subjects resulted from overconsumption of calories that shifted from plant-based foods to animal products containing less complex CHO and related phytonutrients. It is still not clear why the diabetic subjects over-consumed calories because they were not growing like the Nile rats, but processed foods were likely increased. There could also have been behavioral epigenetics at play related to *genetic permissiveness* involving taste preferences. This suggests that the data implicating animal fat and protein [62] may have been confounded by the inability to fully control for the reduction in fiber and phytonutrients reflected in their high-fat, animal-product-rich foods. However, this was partially compensated by identifying the tertile (T3) of the highest CHO intake (65:20:15) among the over-consumers, which was characterized by fewer animal products and more plant-based foods, including more carbohydrates containing fiber and phytonutrients, in essence improving the antidiabetic profile of the diet and reducing the diabetes observed.

The Chinese data [62] are also supported by observations from the Nurses’ Health Study [5,6] and more recent epidemiological studies [41,42], where plant-based energy sources were found to be preferable to animal products, especially meats, for preventing T2DM in more than 450,000 subjects from a globally based meta-analysis. However, these latter data could not be evaluated for caloric intake among subgroups stratified by risk of T2DM, making the Chinese study [62] unique and more relevant to the Nile rat experiments.

### 4.6. Highly-Processed Food, Caloric Intake, and T2DM

Recent data indicating that highly-processed foods can result in over-consumption by as much as 25% in a 2-week period [80,81] support both the findings of the Nile rat experiments and the Chinese data [62]. An emphasis on caloric intake as a major risk factor in diabetes risk was supported by clinical data from a month-long metabolic study [81]. That report [81] and related human data [80] came to the same conclusion from widely different data sets, i.e., that highly-processed foods, such as those bagged, boxed, bottled, brewed, canned and consumed in bulk, are apt to exceed the ability of the central nervous system to ‘count calories’ and control energy intake effectively. As such, processed foods confuse the brain and palate into overeating, especially palatable food products that contain sugars, fat, and salt and present a high GI or GLoad coupled with animal saturated fat and protein [72,82].

Moreover, it was not the amount of CHO, fat or protein so much as how the macronutrients were processed prior to consumption that evoked the undesired impact on metabolism [81]. To control for that possibility, the same CHO:fat:protein energy ratio was supplied such that the source and processing of the foods differed, but not the macronutrient component profiles themselves.

This differed from the Nile rat study designs, which provided the same CHO, same fat, and same protein but in varied, controlled amounts. The human subjects were provided the same macronutrient ratios under controlled conditions, but the components differed slightly to allow for processing differences [81]. In addition to processing and food-source differences, more than likely the outcome included an ill-defined behavioral component, as well [84,85,86].

### 4.7. Potential Genes Involved

Additionally, with the alterations in cecum and gut observed in Nile rats [1] and the functional role of intestinal K-cells and L-cells in mind, cellular damage or a decrease in the secretion of GIP, GLP-1, and PYY might contribute to the consumption of extra calories by Nile rats or a human population that preferentially selects highly-processed foods and, in turn, influences T2DM risk [87]. The diet model of ultra-processed foods would seem to be relevant to the Nile rat experiments because the highly processed semipurified diets in the Nile rat studies with a GLoad greater than 175/2000 kcal human equivalents resulted in a greater probability of developing T2DM. Similar to Nile rats, the risk of MetS in humans was increased in conjunction with increasing insulin resistance, elevated leptin, and decreased adiponectin, observations previously reported for Nile rats [88].

It is also noteworthy that male and female indigenous hunter-gatherers in the Hesta tribes of Central Africa reportedly eat the same number of calories as their equivalent North American counterparts (2000–2500 kcal/d) despite extensive exercise related to food gathering. Nonetheless, these nomadic tribes develop no MetS, obesity, or T2DM when consuming their totally unprocessed native foods, mostly based on native plants but including substantial wild-game meats. The main diet difference versus the North American diet, coupled with the extensive exercise required for food gathering, is their advantageous consumption of natural vegetables and fruits containing plant fibers and phytonutrients, which presumably help control their appetite, microbiome and caloric intake and energy utilization [89].

An overarching implication from these studies is that risk for T2DM increases in a population that preferentially selects highly-processed foods that also result in excess caloric intake, especially if the extra calories tend to come from processed foods containing more animal fat and protein along with refined CHO and GLoad, including less fiber and plant-based proteins and phytonutrients, such as polyphenols and related antioxidant compounds [80,81,82,83,84,85,86,87]. The Nile rat data appears to parallel this scenario in terms of caloric intake, but the current data further document the relative risk that accrues when a high GLoad accompanies extra kcal/day intake in a stress-laden social environment of lab animal housing [23,24] that parallels affluence and industrialization in humans [82,84,85,86]. It is possible that L-cells in the small intestine become compromised and no longer secrete adequate PYY, the satiety regulatory hormone [87].

### 4.8. Diurnal Rhythm Compromised

Another interesting aspect of the story that has recently surfaced in terms of MetS and T2DM induction is the disruption of the circadian rhythm in diurnal species. The story is best explained/demonstrated by the Israeli sand rat, which, like the diurnal Nile rat, is a North African desert rodent in the gerbil family that lives in the wild on a high-fiber, low-caloric density plant diet but develops T2DM on a high-density diet in captivity [90].

In the lab environment of the captive state, a fixed 12/12h light-dark cycle disrupts their normal circadian rhythm and metabolic functions, including glucose metabolism that is exacerbated by a hiCHO, calorically-dense diet. Their mental capacity is altered, as well, so they become stressed, characterized by anxiety and depression. Similar to the Nile rat, this distortion is accompanied by high blood pressure, kidney dysfunction, and diabetes. The syndrome can be prevented by conducting the experiments outside the lab environment in natural lighting circumstances, even if a hiCHO diet is continued [24]. A similar story likely pertains to circadian-stressed humans developing T2DM [23].

In essence, what our experiments demonstrate is that this metabolic disruption of the captive diurnal rat, including its diabetes induction, can be modulated by diet, which exposes certain dietary variables that can deter, or even prevent, the underlying syndrome by improving the CHO-fiber content of the diet. In our experiments, the CHO:fat ratio did not prevent the metabolic disease, but it did mitigate the outcome when the CHO load was reduced, and it adds to the notion that the microbiome may be involved with the diurnal disturbance. It also suggests that rapid growth is an added stress during the regulation of normal diurnal activity.

## 5. Conclusions

In summary, the current Nile experiments reassert the relevance of the model for diet-induced diabetes in humans and collectively reveal that the strongest positive correlation between RBG or OGTT outcome and clinical T2DM was linked to caloric intake, coupled secondarily to the diet macronutrient composition per se, which mimics the story described for humans [62,80,81,86].

Associations between animal saturated fat and protein and T2DM risk derived from epidemiological studies may be more related to the simultaneous lack of plant-based fiber and related antioxidants and other phenolic compounds, or both in combination. Thus, the lack of plant-based foods, in general, may be at fault, while saturated fat and red meat may largely represent animal products as foods that lack both fiber and phytonutrients. Further work is needed to explore protein sources and composition, fiber content and type, and plant antioxidants/phenolics in well-designed studies that allow one to distinguish between these confounding nutritional variables and T2DM induction.

This is the third Nile rat report focused on diet macronutrient composition that illustrates the importance of *genetic permissiveness* coupled with caloric intake and the GLoad during induction of T2DM and emphasizes the need to examine the genetics involving the CNS glucose-regulatory aspects that affect food intake control. Diet-related control implicitly may relate to leptin, adiponectin, and intestinal L cells (FGF-1, PPY, GLP-1, FGF-15, TGR5), K cells (GIP), or a combination of these genes and their hormone products governing food intake. The hormones themselves are potentially impacted by diet composition and may be influencing whole-body energy dynamics [87,91]. The overall scenario reflects the central role of the CNS-hypothalamic ventromedial nucleus during the regulation of both glucose and general energy homeostasis, including adipose tissue dynamics centered around whole-body thermoregulation orchestrated by the microbiome [72,74].

The implication is that *genetic permissiveness*, expressed early on in neonatal Nile rats in artificial housing conditions that adversely affect their circadian rhythm, may distort metabolism. This distortion results in greater food consumption and caloric intake by individual rats to impact growth rate as early as the first weeks of feeding in the post-weaning period. The model concurs with the hypothesis that the origins of T2DM may actually start with metabolic/emotional distress affecting food intake control and diet selection behaviors formed in utero or early childhood that disrupt the microbiome and energy intake and metabolism, especially involving glucose and fatty acids [1].

Consequently, dietary CHO results in chronic hyperglycemia most aggressively in genetically *susceptible* rats that overeat a hiCHO diet that compromises their glucose metabolism. It is unclear whether *resistant* rats uniquely express genes and a microbiome that protects against overeating and glucose over-absorption or whether *susceptible* rats lack protective genetic or epigenetic mechanisms to cope with the dietary glucose overload.

## Supplementary Materials

The following supporting information can be downloaded at: https://www.mdpi.com/article/10.3390/nu14153064/s1, Table S1. Diet Compositions; Table S2. Fatty Acid Profile of the Fat in each Diet; Table S3: Diabetic assessment of 3 week old male Nile rats fed PKOB diets with different CHO:fat ratios and GLoads for 10-weeks (Experiment 1)—Unsplit by T2DM; Table S4: Diabetic assessment of 3 week old male Nile rats fed AFB diets with different CHO:fat ratios and GLoads for 10 weeks (Experiment 2)—Unsplit by T2DM; Table S5: Diabetic assessment of 3 week old male Nile rats fed PKOB diets with different CHO:fat ratios and GLoads for 10 weeks (Experiment 1)—Split into *resistant* or *susceptible* to T2DM based on RBG; Table S6: Diabetic assessment of 3 week old male Nile rats fed AFB diets with different CHO:fat ratios and GLoads for 10 weeks (Experiment 2)—Split into resistant or susceptible to T2DM; Table S7: Diabetic assessment of 3 week old male Nile rats fed PKOB diets with different CHO:fat ratios and Gloads for 10 weeks sorted into Quintiles of Average kcal/day (Experiment 1)—Unsplit; Table S8: Diabetic assessment of 3 week old male Nile rats fed AFB diets with different CHO:Fat ratios and GLoads for 10 weeks sorted into Quintiles of Average kcal/day (Experiment 2)—Unsplit; Table S9: Diabetic assessment of 3 week old male Nile rats fed PKOB diets with different CHO:fat ratios and GLoads for 10 weeks (Experiment 1)—sorted into Quintiles of Average kcal/day and split into resistant or susceptible to T2DM; Table S10: Diabetic assessment of 3 week old male Nile rats fed AFB diets with different CHO:fat ratios and GLoads for 10 weeks (Experiment 2)—sorted into Quintiles of Average kcal/day and split into *resistant* or *susceptible* to T2DM; Table S11: Diabetic assessment of 3 week old male Nile rats fed PKOB diets with different CHO:fat ratios and GLoads for 10 weeks sorted into Quintiles of 10week RBG (Experiment 1); Table S12: Diabetic assessment of 3 week old male Nile rats fed AFB diets with different CHO:fat ratios and GLoads for 10 weeks sorted into Quintiles of 10 weeks RBG (Experiment 2).
