# Supplementary material for: Dietary Carbohydrate as Glycemic Load, Not Fat, Coupled with Genetic Permissiveness Favoring Rapid Growth and Extra Calories, Dictate Metabolic Syndrome and Diabetes Induction in Nile Rats (Arvicanthis niloticus)
Source: Nutrients. 2022 Jul 26;14(15):3064. doi: 10.3390/nu14153064 (PMC9331090; doi:10.3390/nu14153064)
Supplement: Supplementary file 1 [file nutrients-14-03064-s001.zip › nutrients-1792724-supplementary.pdf]

nutrients

Supplementary Materials

Table S1. Diet Compositions.

	Diet				
**Experiment 1 (PKOB) Diet#**	**195**	**196**	**197**	198	199
**Experiment 2 (AFB) Diet#**	200	201	202	203	204
CHO:fat:protein %energy	70:10:20	60:20:20	50:30:20	40:40:20	20:60:20
CHO/Fat %energy ratio	7.0	3.0	1.7	1.0	0.3
kcal/g as dry weight	4.0	4.2	4.5	4.8	5.5
Gload (units/2000 kcal)	293	252	210	167	84
**Ingredient**			**g/kg**		
**Protein**					
Casein	100	106	112	120	138
Lactalbumin	100	106	112	120	138
**CHO**					
Dextrose	349	315	281	238	138
Cornstarch	288 (+60 gel)*	256 (+60 gel)*	222 (+60 gel)*	178 (+60 gel)*	78 (+60 gel)*
**Fat**					
Experiment 1 – PKOB**[a]	44	95	148	214	369
Experiment 2 – AFB (%fat)***[b]	46 (44)	100 (95)	156 (148)	225 (214)	388 (369)
Mineral mix (Ausman-Hayes)[c]	44	47	49	53	61
Vitamin mix (Hayes-Cathcart)[c]	11	12	12	13	15
**Choline Chloride**	3	3	3	3	3
**Cholesterol** – Experiment 1	0.6	0.6	0.6	0.6	0.6
**Cholesterol** – Experiment 2	0.6 (+0.05)***	0.6 (+0.10)***	0.6 (+0.16)***	0.6 (+0.23)***	0.6 (+0.41)***

[a]PKOB composition: 31% Palm Kernel Oil, 33% Soybean Oil, 36% Canola oil; [b]AFB composition: 21% Butter, 46% beef tallow, 15% lard,18% Soybean oil; [c]Leow et al. (2016) [40]; *60g cornstarch added to 800mL water to form gel; **Pre-made PKOB had 20% water content while PKOB formulated in the lab is 100% fat; ***AFB is adjusted to butter having 20% water with cholesterol contributed by animal fats.

Table S2. Fatty Acid Profile of the Fat in each Diet.

				Diet	
**Experiment**				**Experiment 1**	**Experiment 2**
**Fat Composition**				**PKOB[1]**	**AFB[2]**
**Saturated fatty acid profile^**				**Fatty acid Percentage (%)**	
Acetic Acid + Propionic Acid			2:0+3:0	n/a	n/a
Butyric Acid + Caproic Acid			4:0+6:0	0.1	1.2
Caprylic Acid			8:0	3.3	0.3
Capric Acid			10:0	1.1	0.5
Lauric Acid			12:0	14.9	0.7
Myristic Acid			14:0	5.1	4
Palmitic Acid			16:0	7.5	22.6
Stearic Acid			18:0	2.8	13.8
**Unsaturated fatty acid profile**					
Oleic acid	n9**	18:1(n-9)		34.0	36.3
Palmitoleic acid	n7**	16:1(n-7)		2.5	2.6
Linoleic acid	n6**	18:2(n-6)		26.7	13.0
α-Linoleic acid	n3**	18:3(n-3)		5.7	1.8
Total saturated fatty acids				32.5	44.7
Total monounsaturated fatty acids				34.1	40.2
Total polyunsaturated fatty acids				33.5	14.8
P/S* ratio				1.00	0.35
S:M:P in diet				33:34:33	9:8:3
**CHO:fat:protein %energy ratio**				**18:2(n-6)****	**(total polyunsaturated fatty acid) %energy**
70:10:20				2.7 (3.4)	1.3 (1.5)
60:20:20				5.3 (6.7)	2.6 (3.0)
50:30:20				8.0 (10.1)	3.9 (4.4)
40:40:20				10.7 (13.4)	5.2 (5.9)
20:60:20				16.0 (20.1)	7.8 (8.9)

[1]PKOB = 31% Palm Kernel Oil, 33% Soybean Oil, 36% Canola Oil. S:M:P ratio is balanced to 1:1:1 with an ideal P/S ratio of 1.0; [2]AFB = 21% Butter, 46% Tallow, 15% Lard, 18% Soybean oil. S:M:P ratio is 9:8:3. with P/S ratio of 0.35 typical of saturated fat in the American diet; *18:2+18:3 /8:0-18:0; ** n3, n6, n7 and n9 refer to omega 3, 6, 7 and 9, respectively. ^Diet fatty acids were calculated from published data on fatty acid compositions of fats and oils. [see USDA Handbook 8. Food compositions] and diet compositions in Table S1.

Table S3. Diabetic assessment of 3week old male Nile rats fed PKOB diets with different CHO:fat ratios and GLoads for 10 weeks (Experiment 1) – Unsplit by T2DM.

	Diet				
**Diet#**	**195**	**196**	**197**	**198**	**199**
**n=**	**14**	**14**	**13**	**14**	**14**
**CHO:fat:protein %energy**	**70:10:20**	**60:20:20**	**50:30:20**	**40:40:20**	**20:60:20**
**P/S ratio**	1.00	1.00	1.00	1.00	1.00
**Resistant/susceptible (% susceptible)**					
10 week RBG <75mg/dl>	8/6 (43%)	5/9 (64%)	7/6 (46%)	9/5 (36%)	11/3 (21%)
10 week 30′ OGTT <175mg/dl>	6/8 (57%)	2/12 (86%)	6/7 (54%)	6/8 (57%)	11/3 (21%)
10 week FBG <60mg/dl>	12/2 (14%)	10/4 (29%)	9/4 (31%)	7/7 (50%)	8/6 (43%)
**kcal/g**	4.0	4.2	4.5	4.8	5.5
**CHO:fat %energy ratio**	7	3	1.7	1	0.3
**Fat:protein %energy ratio**	0.5	1.0	1.5	2.0	3.0
**GLoad (g/2000 kcal)**	280	245	204	162	82
**RBW (g)**					
Initial (3 weeks of age)	31±6	31±3	32±6	31±3	32±4
After 3 weeks	70±8	72±6	74±8	71±5	69±8
After 6 weeks	85±8	91±8	90±11	88±8	88±10
After 10 weeks	97±8^{a}	106±9^{a}	104±12	104±9	103±11
**BW gain/day (g/day)**	0.88±0.12^{a}	1.00±0.12^{a}	0.95±0.15	0.97±0.12	0.97±0.19
**Energy intake**					
g/day	16.0±3.0^{abcd}	14.5±0.7^{aef}	13.9±1.6^{bgh}	12.1±0.7^{cegi}	10.5±0.7^{dfhi}
**kcal/day**					
week 2	25±4	26±2	27±2	24±2	26±2
week 8	28±8	27±3	28±4	24±1	26±2
10 week average	26±5	26±1	27±3	24±1	26±2
**kcal/kg BW /10 week**	265±44	247±20	259±26	227±12	250±20
**18:2(n-6) %energy**	2.7	5.3	8.0	10.7	16.0
**GLoad**					
/rat/day	3.6±0.7^{abcd}	3.2±0.2^{aefg}	2.7±0.3^{behi}	1.9±0.1^{cfhj}	1.1±0.1^{dgij}
/kg BW at 10 weeks	37±6 ^{abcd}	30±2 ^{aefg}	26±3 ^{behi}	18±1 ^{cfhj}	10±1 ^{dgij}
Cumulative, 10 weeks	253±48 ^{abcd}	223±11 ^{aefg}	191±22 ^{behi}	134±7 ^{cfhj}	74±5 ^{dgij}
cumGLoad/rat/kcal	0.14 ^{abcd}	0.12 ^{aefg}	0.10 ^{behi}	0.08 ^{cfhj}	0.04 ^{dgij}
**10th Week Water Intake (ml)**	81±158	57±56	78±80	36±15	40±10
**Food efficiency (g BW gained/1000 kcal)**					
1-6 weeks	46±8	50±6	46±5	50±8	46±6
1-10 weeks	35±6	38±4	36±4	41±5	37±6
**RBG (mg/dl)**					
After 6 weeks	190±174^{abc}	101±68^{a}	129±124	71±19^{b}	66±12^{c}
After 10 weeks	144±167	159±126^{a}	168±165^{b}	110±80	64±10^{ab}
Severity (> 75mg/dl)	+69	+89	+93	+35	-11
**OGTT (BG mg/dl)**					

**After 6 weeks**					
FBG, 0 min	57±18	60±24	52±15	53±12	53±9
30 min	268±129	292±95^{a}	250±137	227±62	201±60^{a}
**After 10 weeks**					
FBG, 0 min	48±9^{a}	55±9	54±19	60±18	62±21^{a}
30 min	213±101	279±152^{a}	245±150	209±85	172±51^{a}
60 min	147±109	183±114	202±153	190±92	151±58
**Organ weight (%BW)**					
Liver	3.7±0.7	3.6±0.5	3.8±0.7	3.4±0.4	3.6±0.4
Kidney	0.8±0.2^{abc}	0.7±0.1^{a}	0.7±0.1	0.7±0.1^{b}	0.7±0.1^{c}
Cecum	1.3±0.5^{abcd}	1.0±0.2^{a}	1.1±0.3^{b}	1.0±0.1^{c}	1.1±0.2^{d}
Adipose					
Epididymal	2.4±0.7^{abc}	2.9±0.5^{a}	2.9±0.7	3.1±0.4^{b}	3.3±0.8^{c}
Perirenal	1.4±0.6^{a}	1.9±0.5	1.8±0.5	2.0±0.8^{a}	1.6±0.5
BAT	1.8±0.6^{ab}	2.4±0.5^{ac}	2.2±0.6^{b}	2.0±0.4	1.9±0.5^{c}
Total fat	5.6±1.7^{abcd}	7.2±0.9^{a}	6.9±1.1^{b}	7.1±1.3^{c}	6.9±1.4^{d}
Carcass	74±2	74±2	74±1	74±2	73±2
**Body length (cm)**	13.3±0.3	13.4±0.3	13.4±0.4	13.3±0.4	13.4±0.7
**BMI (kg/m^2)**	5.4±0.4	5.7±0.4	5.6±0.5	5.7±0.4	5.5±0.5
**Plasma**					
TC (mg/dl)	103±42	112±25	96±18	94±21	96±17
TG (mg/dl)	73±26^{a}	87±33^{bc}	79±21^{d}	66±17^{b}	53±10^{acd}

Values are mean±SD (n=13–14); $^{a,b,c.}$ Means in a row sharing a common superscript differ ($p<0.05$) by by one-way ANOVA and Fisher's PLSD test.

Table S4. Diabetic assessment of 3 week old male Nile rats fed AFB diets with different CHO:fat ratios and GLoads for 10 weeks (Experiment 2) – Unsplit by T2DM.

	Diets				
**Diet #**	**200**	**201**	**202**	**203**	**204**
**n=**	14	14	14	13	14
**CHO:fat:protein %energy**	70:10:20	60:20:20	50:30:20	40:40:20	20:60:20
**P/S ratio**	0.35	0.35	0.35	0.35	0.35
**Resistant/suscepible (% susceptible)**					
10 week RBG <75mg/dl>	10/4 (29%)	10/4 (29%)	7/7 (50%)	11/2 (15%)	11/3 (21%)
10 week 30′ OGTT <175mg/dl>	10/4 (29%)	7/7 (50%)	6/8 (57%)	8/5 (38%)	7/7 (50%)
10 week FBG <60mg/dl>	12/2 (14%)	8/6 (43%)	9/5 (36%)	10/3 (23%)	10/4 (29%)
**kcal/g**	4.0	4.2	4.5	4.8	5.5
**CHO:fat ratio**	7	3	1.7	1	0.3
**Fat:protein %energy ratio**	0.5	1.0	1.5	2.0	3.0
**GLoad (g/2000 kcal)**	280	245	204	162	82
**RBW (g)**					
Initial (3 weeks of age)	32±5	32±4	32±4	31±4	32±6
After 3 weeks	70±6	71±7	72±7	71±9	67±12
After 6 weeks	85±8	87±9	88±9	85±11	84±12
After 10 weeks	98±10	100±12	103±10	99±14	95±14
**BW gain/day (g/day)**	0.87±0.11	0.90±0.14	0.94±0.14	0.89±0.16	0.84±0.15
**Food intake (g BW gained/1000 kcal)**					
g/day	14.3±1.6^{abc}	13.4±1.0^{de}	13.2±1.1^{afg}	11.4±1.0^{bdf}	10.6±1.3^{ceg}
**kcal/day**					
week 2	24±3^{a}	25±2	27±2^{ab}	24±2^{bc}	26±3^{c}
week 8	23±4^{a}	24±3^{b}	25±3^{c}	23±2^{d}	28±4^{abcd}
10 week average	23±3^{ab}	24±2^{c}	26±2^{ad}	22±2^{de}	26±3^{bce}
**kcal/kg BW /10 week**	235±23^{a}	242±22^{b}	250±21^{cd}	226±20^{ce}	277±34^{abde}
**18:2(n-6) %energy**	1.3	2.6	3.9	5.2	7.8
**GLoad**					
/rat/day	3.2±0.4^{abcd}	2.9±0.2^{aefg}	2.6±0.3^{behi}	1.8±0.2^{cfhj}	1.0±0.1^{dgij}
/kg BW at 10weeks	33±3^{abcd}	30±3^{aefg}	25±3^{behi}	18±2^{cfhj}	11±2^{dgij}
Cumulative, 10 weeks	225±25^{abcd}	206±16^{aefg}	180±20^{behi}	126±11^{cfhj}	71±9^{dgij}
cumGLoad/ rat/kcal	0.14 ^{abcd}	0.12 ^{aefg}	0.10 ^{behi}	0.08 ^{cfhj}	0.04 ^{dgij}
**10th Week Water Intake (ml)**	46±37	29±10	42±30	32±6	38±20
**Food efficiency (g BW gained/1000 kcal)**					
1-6 weeks	47±5	48±5^{a}	45±6^{b}	50±8^{bc}	43±7^{ac}
1-10 weeks	38±5^{a}	38±4^{b}	37±6^{c}	40±5^{d}	32±5^{abcd}
**RBG (mg/dl)**					
After 6 weeks	102±111	73±25	71±26	64±17	74±60
After 10 weeks	**120±113**	**86±67^{a}**	**167±172^{abc}**	**66±12^{b}**	**78±62^{c}**
Severity (> 75mg/dl)	+45	+11	+92	-9	+3
**OGTT (BG mg/dl)**					

**After 6 weeks**					
FBG, 0 min	48±11	48±12	48±7	47±14	54±16
30 min	213±116	187±82	220±82	185±59	225±89
**After 10 weeks**					
FBG, 0 min	54±30	55±14	54±13	49±18	57±23
30 min	177±127	192±105	231±106	165±73	196±71
60 min	127±76	105±63	184±135	127±81	185±148
**Organ weight (%BW)**					
Liver	3.5±0.7	3.4±0.4	3.6±0.6	3.2±0.3	3.5±0.4
Kidney	0.9±0.6	0.7±0.1	0.7±0.1	0.7±0.1	0.8±0.1
Cecum	1.1±0.3	1.1±0.1	1.1±0.2	1.1±0.2	1.2±0.4
Adipose					
Epididymal	2.7±0.5	2.5±0.5	2.9±0.7	2.7±0.7	2.7±0.9
Perirenal	1.6±0.5	1.7±0.4	1.8±0.3	1.6±0.5	1.4±0.5
BAT	2.1±0.7	2.1±0.5	2.2±0.5	2.1±0.6	1.8±0.7
Total fat	6.4±1.5	6.3±1.2	6.9±1.2	6.5±1.5	6.0±1.7
Carcass	74±2	74±2	74±2	75±2	74±2
**Body length (cm)**	13.3±0.8	13.2±0.3	13.3±0.3	13.3±0.7	13.2±0.5
**BMI (kg/m²)**	5.38±0.47	5.52±0.56	5.69±0.56	5.35±0.51	5.32±0.48
**Plasma**					
TC (mg/dl)	127±40^{a}	119±29^{b}	121±20^{c}	98±11^{abcd}	120±19^{d}
TG (mg/dl)	98±47^{a}	93±26^{b}	135±74^{abcd}	75±20^{c}	72±30^{d}

Values are mean±SD (n=13-14); ^{a,b,c} Means in a row sharing a common superscript differ (p<0.05) by by one-way ANOVA and Fisher's PLSD test.

Table S5. Diabetic assessment of 3 week old male Nile rats fed PKOB diets with different CHO:fat ratios and GLoads for 10 weeks (Experiment 1) – Split into resistant or susceptible to T2DM based on RBG.

	Diet									
**Diet #**	**195**		**196**		**197**		**198**		**199**	
**n=**	**14**		**14**		**14**		**14**		**14**	
**CHO:fat:protein %energy**	**70:10:20**		**60:20:20**		**50:30:20**		**40:40:20**		**20:60:20**	
**P/S Ratio**	1.00		1.00		1.00		1.00		1.00	
**Resistant/susceptible (% susceptible)**										
10 week RBG <75mg/dl>	8/6 (43%)		5/9 (64%)		7/6 (46%)		9/5 (36%)		11/3 (21%)	
10 week 30′OGTT <175mg/dl>	6/8 (57%)		2/12 (86%)		6/7 (54%)		6/8 (57%)		11/3 (21%)	
10 week FBG <60mg/dl>	12/2 (14%)		10/4 (29%)		9/4 (31%)		7/7 (50%)		8/6 (43%)	
**kcal/g**	4.0		4.2		4.5		4.8		5.5	
**CHO:fat %energy ratio**	7.0		3.0		1.7		1.0		0.3	
**Fat:protein %energy ratio**	0.5		1		1.5		2		3	
**GLoad/2000 kcal**	280		245		204		162		82	
	**Data Split into *resistant* and *susceptible* rats**									
	**Resist**	**Suscept**	**Resist**	**Suscept.**	**Resist.**	**Suscept.**	**Resist.**	**Suscept.**	**Resist.**	**Suscept.**
**Kcal/day**	24±3	28±7	25±1	27±1	25±2	29±3	23±1	25±1	26±2	25±1
**10 week RBG (mg/dl)**	59±12	257±213	63±4	212±131	61±7	293±176	62±8	197±76	60±7	79±2
**n=**	**6**	**8**	**5**	**9**	**7**	**6**	**9**	**5**	**11**	**3**
**RBW (g)**										
Initial (3 weeks of age)	31±6	32±6	32±2	31±3	30±5	35±6	30±3	32±4	33±5	30±3
After 3 weeks	69±7	71±10	71±5	73±7	70±8	79±4	70±5	74±4	70±8	64±2
After 6 weeks[a]	86±9	84±8	86±8	94±7	85±11	96±9	84±6	94±6	90±10	81±8
After 10 weeks	97±8	97±9	101±7	109±9	100±12	109±11	100±7	112±6	105±11	96±6
**BW gain/day (g/day)**	0.88±0.1	0.87±0.1	0.92±0.1	1.04±0.1	0.92±0.1	0.98±0.2	0.92±0.1	1.06±0.1	1.00±0.1	0.85±0.1
**Energy intake**										
g/day[bc]	15.0±1.6	17.4±4.1	14.0±0.4	14.9±0.6	12.9±1.2	15.0±1.3	11.8±0.6	12.6±0.5	10.6±0.7	10.0±0.2
**kcal/day**										
week 2[b]	23±2	25±3	25±1	25±2	26±3	28±1	23±2	24±1	26±2	25±1
week 8	23±3	27±6	25±3	25±1	24±2	28±3	23±3	25±1	27±3	24±2
10 week average[bc]	24±3	28±7	25±1	27±1	25±2	29±3	23±1	25±1	26±2	25±1
**kcal/kg BW /10 week[b]**	249±19	287±60	248±11	246±24	251±18	268±32	232±11	219±8	249±22	256±9
**18:2(n-6) %energy**	2.7		5.3		8		10.7		16	
**GLoad**										
/rat/day[bc]	3.4±0.3	3.9±0.9	3.1±0.1	3.3±0.1	2.5±0.2	2.9±0.2	1.9±0.1	2.0±0.1	1.1±0.1	1.0±0.0
/kg BW at 10 weeks[b]	35±3	40±8	30±1	30±3	26±2	27±3	19±1	18±1	10±1	11±0
Cumulative, 10 weeks[bc]	237±25	274±64	215±7	228±10	178±16	206±18	131±7	139±5	75±5	70±2
cumGLoad/rat/kcal	0.14	0.14	0.12	0.12	0.1	0.1	0.08	0.08	0.04	0.04
**10th Week Water Intake (ml)[c]**	37±15	140±239	28±10	73±65	36±13	126±100	33±6	40±26	41±8	37±17
**Food efficiency (g BW gained/1000 kcal)**										
1-6 weeks	49±6	43±8[a]	45±5	52±5[a]	46±5	45±6	52±7	48±10	46±5	44±9
1-10 weeks[b]	37±4	32±7	36±4	39±4	37±3	34±6	40±5	43±3	38±6	34±3
**RBG (mg/dl)**										

After 6 weeks[bc]	141±102	254±236	77±18	114±83	71±8	198±162	70±21	74±17	66±10	65±21
After 10 weeks[c]	**59±12**	**257±213**	**63±4**	**212±131**	**61±7**	**293±176**	**62±8**	**197±76**	**60±7**	**79±2**
Severity (mg/dl > 75)	-16	+182	-12	+137	-14	+217	-13	+125	-15	+4
**After 6 weeks**										
FBG, 0 min	56±18	57±19	54±7	64±29	47±8	58±20	50±9	58±15	53±9	50±7
30 min[c]	216±86	336±151	244±34	319±109	175±57	338±156	205±57	266±56	215±59	149±16
**After 10 weeks**										
FBG, 0 min[bc]	48±12	47±6	53±8	57±10	49±20	60±17	58±17	63±21	57±13	82±37
30 min[c]	165±80	278±95	178±11	336±166	176±56	326±188	170±53	278±91	175±58	161±13
60 min[c]	120±120	182±88	95±41	233±113	116±44	302±177	160±71	244±107	148±58	158±66
**Organ weight (%BW)**										
Liver[c]	3.5±0.4	4.0±0.9	3.5±0.1	3.7±0.7	3.5±0.5	4.2±0.9	3.2±0.3	3.7±0.4	3.7±0.4	3.2±0.2
Kidney[b]	0.7±0.1	0.9±0.3	0.7±0.1	0.7±0.1	0.7±0.1	0.8±0.1	0.7±0.0	0.7±0.1	0.7±0.1	0.7±0.1
Cecum[b]	1.2±0.1	1.5±0.7	1.0±0.1	1.1±0.2	1.1±0.2	1.1±0.4	1.1±0.1	1.0±0.1	1.1±0.2	1.0±0.1
Adipose										
Epididymal[b]	2.5±0.5	2.3±0.9	3.2±0.5	2.8±0.4	2.8±0.6	3.0±1.0	3.1±0.4	3.2±0.5	3.4±0.9	3.2±0.8
Perirenal[ab]	1.5±0.6	1.4±0.7	1.8±0.4	1.9±0.5	1.7±0.5	1.9±0.5	1.6±0.5	2.7±0.6	1.7±0.5	1.3±0.1
BAT[b]	1.9±0.6	1.6±0.6	2.2±0.5	2.4±0.5	2.1±0.5	2.3±0.8	1.8±0.3	2.3±0.4	2.0±0.6	1.7±0.4
Total fat[b]	5.8±1.4	5.4±2.1	7.1±1.0	7.2±0.9	6.6±1.0	7.2±1.3	6.4±1.0	8.2±0.7	7.1±1.5	6.2±0.4
Carcass	75±2	74±2	74±2	73±2	74±1	73±1	74±2	73±1	73±2	74±1
**Body length (cm)**	13.3±0.2	13.2±0.5	13.3±0.2	13.4±0.3	13.2±0.3	13.6±0.4	13.3±0.5	13.4±0.4	13.6±0.7	13.0±0.3
**BMI (kg/m^2)**	5.3±0.5	5.4±0.3	5.5±0.5	5.9±0.3	5.5±0.5	5.7±0.5	5.5±0.3	6.0±0.2	5.5±0.5	5.5±0.4
**Plasma**										
TC (mg/dl)[a,c,d]	84±14	128±55	96±16	121±26	91±9	101±25	102±20	79±13	95±17	101±22
TG (mg/dl)[a,c,d]	69±17	78±35	65±15	100±33	71±15	88±26	59±14	79±16	54±10	50±13

Values are mean±SD (n=3-11); [a]Significant (P < 0.05) interaction term for diet x diabetes class (RBG <75 mg/dl>) revealed by two-way ANOVA; [b]Significant (P < 0.05) effect of diet (GLoad) by two-way ANOVA; [c]Significant (P < 0.05) increase due to diabetes (RBG >75 mg/dl) by two-way ANOVA; [d]Significant (P < 0.05) decrease due to diabetes (RBG >75 mg/dl) by two-way ANOVA.

Table S6. Diabetic assessment of 3 week old male Nile rats fed AFB diets with different CHO:fat ratios and GLoads for 10 weeks (Experiment 2) – Split into resistant or susceptible to T2DM.

	Diets									
**Diet #**	**200**		**201**		**202**		**203**		**204**	
**n=**	**14**		**14**		**14**		**13**		**14**	
**CHO:fat:protein %en**	70:10:20		60:20:20		50:30:20		40:40:20		20:60:20	
**P/S ratio**	0.35		0.35		0.35		0.35		0.35	
**Resistant/susceptible (% susceptible)**										
10 week RBG <75mg/dl>	10/4 (29%)		10/4 (29%)		7/7 (50%)		11/2 (15%)		11/3 (21%)	
10 week 30′OGTT <175mg/dl>	10/4 (29%)		7/7 (50%)		6/8 (57%)		8/5 (38%)		7/7 (50%)	
10 week FBG <60mg/dl>	12/2 (14%)		8/6 (43%)		9/5 (36%)		10/3 (23%)		10/4 (29%)	
**kcal/g**	4.0		4.2		4.5		4.8		5.5	
**CHO:fat %en ratio**	7.0		3.0		1.7		1.0		0.3	
**Fat:protein %en ratio**	(0.5)		(1.0)		(1.5)		(2.0)		(3.0)	
**GLoad/2000 kcal**	280		245		204		162		82	
	**Data Split into *resistant* and *susceptible* rats**									
	**Resist**	**Suscept**	**Resist**	**Suscept**	**Resist**	**Suscept**	**Resist**	**Suscept**	**Resist**	**Suscept**
**kcal/day**	22±2	25±3	23±2	26±1	25±2	26±2	22±2	23±0	26±3	**27±5**
**10 week RBG (mg/dl)**	63±9	262±132	57±8	160±96	58±7	275±192	62±6	89±6	58±9	151±121
**n=**	**10**	**4**	**10**	**4**	**7**	**7**	**11**	**2**	**11**	**3**
**RBW (g)**										
Initial(3 weeks old)[c]	31±4	34±5	32±4	32±4	31±3	33±4	30±4	37±0	30±6	37±4
After 3 weeks[c]	68±6	74±5	69±7	74±3	70±6	73±9	70±9	77±5	64±10	77±15
After 6 weeks[c]	82±8	93±3	85±9	93±6	87±8	88±10	83±11	93±10	80±9	98±13
After 10 weeks[c]	95±10	107±6	96±11	109±7	103±10	103±10	97±15	109±11	92±11	108±21
**BW gain/day (g/day)**	0.8±0.1	1.0±0.1	0.9±0.1	1.0±0.1	1.0±0.1	0.9±0.1	0.9±0.1	0.9±0.1	0.8±0.1	0.9±0.2
**Food intake**										
g/day[bc]	13.7±1.1	15.7±1.7	13.0±0.9	14.4±0.5	12.8±1.0	13.7±0.9	11.3±1.1	11.8±0.2	10.5±1.2	**11.0±1.9**
**kcal/day**										
week 2[c]	23±3	26±1	25±3	26±1	26±2	28±2	23±2	25±2	26±3	**27±4**
week 8[bc]	22±2	26±4	23±3	26±3	24±3	26±3	23±3	24±0	28±4	**29±6**
10 week average[bc]	22±2	25±3	23±2	26±1	25±2	26±2	22±2	23±0	26±3	**27±5**
**kcal/kg BW/10 week**[b]	234±17	238±36	244±23	238±20	240±21	259±18	228±20	213±17	283±33	**253±34**
**18:2(n-6) %energy**	1.5		3		4.5		6		9	
**GLoad**										
/rat/day[bc]	3.1±0.3	3.5±0.4	2.9±0.2	3.2±0.1	2.5±0.3	2.7±0.2	1.8±0.2	1.9±0.0	1.0±0.1	1.0±0.1
/kg BW at 10 weeks[b]	33±2	33±5	30±3	29±2	24±3	26±2	18±2	17±1	11±1	9±2
Cumulative, 10weeks[bc]	216±18	248±27	199±14	221±8	171±23	189±13	125±12	131±2	72±9	68±7
cumGLoad/rat/kcal	0.14	0.14	0.12	0.12	0.10	0.10	0.08	0.08	0.04	0.04
**10th Week Water Intake (ml)**[c]	34±14	77±60	29±9	30±11	30±14	54±38	31±5	35±11	33±13	52±35

Food efficiency (g BW gained/1000 kcal)

1- 6 weeks	46±4	49±8	48±6	48±5	47±5	43±7	50±8	50±7	41±7	49±3
1-10 weeks	38±4	38±9	37±5	39±4	39±5	34±5	40±5	40±6	32±5	34±6
**RBG (mg/dl)**										
After 6 weeks[abc]	64±13	199±188	68±20	88±35	61±13	82±33	65±18	59±5	60±9	125±135
After 10 weeks[abc]	**63±9**	**262±132**	**57±8**	**160±96**	**58±7**	**275±192**	**62±6**	**89±6**	**58±9**	**151±121**
Severity (mg/dl >75)	-12	+87	-18	+85	-17	+200	-13	+14	-17	+76
**OGTT (BG mg/dl)**										
**After 6 weeks**										
FBG, 0 min[c]	44±9	56±13	49±13	47±11	45±7	51±5	45±14	58±11	52±16	63±18
30 min[c]	167±46	327±168	161±56	253±107	187±66	253±88	181±56	206±97	197±37	328±156
**After 10 weeks**										
FBG, 0 min[c]	44±13	78±49	53±11	59±21	54±18	54±8	44±15	74±22	56±26	62±5
30 min[c]	121±48	316±165	167±63	253±170	180±60	282±122	151±60	240±126	172±37	283±108
60 min[bc]	101±35	194±115	93±32	135±113	124±71	244±161	105±47	246±150	135±67	370±233
**Organ weight (%BW)**										
Liver[c]	3.4±0.3	4.0±1.1	3.3±0.2	3.7±0.5	3.2±0.2	4.0±0.7	3.3±0.3	3.1±0.2	3.5±0.3	3.9±0.8
Kidney	0.9±0.7	0.8±0.2	0.7±0.1	0.8±0.2	0.7±0.0	0.8±0.1	0.8±0.2	0.6±0.1	0.7±0.0	0.8±0.1
Cecum	1.1±0.2	1.2±0.4	1.1±0.1	1.0±0.2	1.0±0.1	1.1±0.2	1.01±0.2	1.0±0.2	1.1±0.4	1.2±0.6
Adipose										
Epididymal	2.7±0.6	2.6±0.4	2.6±0.6	2.5±0.1	2.8±0.4	3.0±1.0	2.6±0.7	3.2±0.0	2.5±0.8	3.5±1.1
Perirenal[c]	1.5±0.5	1.9±0.5	1.6±0.5	1.9±0.2	1.7±0.4	1.8±0.3	1.5±0.3	2.4±0.1	1.3±0.4	2.0±0.5
BAT[c]	1.9±0.6	2.5±0.7	1.9±0.4	2.6±0.3	2.4±0.6	2.1±0.5	2.0±0.6	2.7±0.3	1.6±0.5	2.5±0.9
Total fat[c]	6.1±1.5	7.0±1.5	6.1±1.3	6.9±0.3	6.9±1.3	6.9±1.1	6.1±1.3	8.4±0.3	5.4±1.4	8.0±0.8
Carcass[d]	75±2	72±1	75±2	74±1	74±1	74±3	75±2	74±1	75±2	72±1
**Body length (cm)**[c]	13.1±0.9	13.6±0.6	13.1±0.3	13.5±0.1	13.1±0.2	13.4±0.4	13.2±0.6	14.4±0.2	13.1±0.4	13.5±0.5
**BMI (kg/m^2)**	5.3±0.5	5.6±0.5	5.4±0.6	5.7±0.3	5.8±0.6	5.5±0.5	5.4±0.5	5.1±0.3	5.2±0.4	5.6±0.8
**Plasma**										
TC (mg/dl)[c]	116±26	151±59	113±19	136±44	113±21	129±17	99±12	94±4	116±19	132±10
TG (mg/dl)[bc]	75±19	151±51	84±25	116±11	97±27	172±89	70±17	102±7	64±24	101±34

Values are mean±SD (n=2-11); [a]Significant (P < 0.05) interaction term for diet x diabetes class (RBG <75 mg/dl>) revealed by two-way ANOVA; [b]Significant (P < 0.05) effect of diet (GLoad) by two-way ANOVA; [c]Significant (P < 0.05) increase due to diabetes (RBG >75 mg/dl) by two-way ANOVA; [d]Significant (P < 0.05) decrease due to diabetes (RBG >75 mg/dl) by two-way ANOVA.

Table S7. Diabetic assessment of 3 week old male Nile rats fed PKOB diets with different CHO:fat ratios and GLoads for 10 weeks sorted into Quintiles of Average kcal/day (Experiment 1) – Unsplit by T2DM.

	**Quintiles (kcal/day)**				
**Q_{kcal}**	**1**	**2**	**3**	**4**	**5**
**n=**	**13**	**14**	**14**	**14**	**14**
**kcal/day**	22.2±1.1	24.1±0.3	25.1±0.3	26.4±0.6	29.8±3.0
**P/S Ratio**	**1.00**	**1.00**	**1.00**	**1.00**	**1.00**
**Resistant/susceptible (% susceptible)** 10 week RBG <75mg/dl>	11/2 (15%)	9/5 (36%)	8/6 (43%)	8/6 (43%)	4/10 (71%)
10 week 30′ OGTT <175mg/dl>	11/2 (15%)	9/5 (36%)	6/8 (57%)	4/10 (71%)	1/13
10 week FBG <60mg/dl>	10/3 (30%)	10/4 (29%)	8/6 (43%)	11/3 (21%)	8/6 (43%)
**Diet Code**	5, 0, 1, 7, 0	2, 2, 2, 4, 4	2, 4, 2, 1, 5	1, 6, 2, 2, 3	4, 2, 6, 0, 2
**CHO:fat Score**	3.4±3.0	2.0±2.3	2.3±2.3	2.2±1.8	3.2±2.6
**est. CHO:fat:protein %energy**	63:17:20	60:20:20	56:24:20	56:24:20	51:29:20
**est. GLoad/2000 kcal**	227±55[a]	196±76	191±64	198±68	166±78[a]
**RBW (g)**					
Initial (3 weeks of age)	31±4	32±5	31±4	31±3	34±5
After 3 weeks	67±6[a]	68±6[b]	70±6[c]	72±7[d]	78±6[abcd]
After 6 weeks	80±8[abc]	84±7[def]	90±7[adg]	92±6[be]	96±7[cfg]
After 10 weeks	94±10[abc]	99±8[de]	105±9[a]	107±8[bd]	109±8[ce]
**BW gain/day (g/day)**	0.86±0.13[abc]	0.90±0.12[de]	0.97±0.13[a]	1.02±0.15[bd]	1.01±0.12[ce]
**Energy intake**					
g/day	12.3±1.2[a]	12.2±1.8[b]	12.7±2.1[c]	13.7±1.7[d]	16.1±3.2[abcd]
**kcal/day**					
week 2	22.4±1.5[abcd]	24.2±1.4[aefg]	25.4±1.5[beh]	26.2±1.5[cfi]	28.4±1.8[dghi]
week 8	23.0±1.6[abc]	25.2±0.9[de]	25.8±1.9[af]	27.4±2.2[bdg]	32.1±6.3[cefg]
10 week average	22.2±1.1	24.1±0.3	25.1±0.3	26.4±0.6	29.8±3.0
**kcal/kg BW /10 week**	237±15[a]	244±21[b]	242±21[c]	249±19[d]	276±44[abcd]
**18:2(n-6) %energy**	7.4±4.0	9.9±4.9	9.5±5.4	8.6±4.6	7.3±4.4
**GLoad**					
/rat/day	2.33±0.62[a]	2.09±0.88[b]	2.26±1.03[c]	2.57±0.91	3.22±1.12[abc]
/kg BW at 10 weeks	25±8	21±9[a]	22±10[b]	24±9	30±12[ab]
Cumulative, 10 weeks	163±44[a]	146±61[b]	159±72[c]	180±64	225±79[abc]
cumGLoad/rat/kcal	0.10	0.09	0.10	0.10	0.09
**10th Week Water Intake (ml)**	29±8[a]	35±12[b]	38±14[c]	51±37[d]	135±162[abcd]
**Food efficiency (g BW gained/1000 kcal)**					
1-6 weeks	49±8	46±7	49±5	47±5	46±7
1-10 weeks	38±4[a]	37±5	39±5[b]	38±6[c]	34±6[abc]
**RBG (mg/dl)**					
After 6 weeks	69±21[a]	74±14[b]	85±26[c]	85±53[d]	240±184[abcd]
After 10 weeks	**64±14[a]**	**107±89[b]**	**83±40[c]**	**120±98[d]**	**264±193[abcd]**
Severity (>75mg/dl)	-11	+32	+8	+45	+189
**OGTT (BG mg/dl)**					

**After 6 weeks**					
FBG, 0 min	56±16	54±12	48±8^{a}	54±13	63±25^{a}
30 min	183±44^{ab}	209±63^{c}	214±74^{d}	268±97^{ae}	358±121^{bcde}
**After 10 weeks**					
FBG, 0 min	49±13	57±15	60±25	53±13	59±11
30 min	139±46^{ab}	193±76^{c}	192±69^{d}	236±105^{ae}	353±142^{bcde}
60 min	115±64^{a}	150±90^{b}	144±70^{c}	186±100^{d}	271±135^{abcd}
**Organ weight (%BW)**					
Liver	3.3±0.3^{a}	3.4±0.3^{b}	3.5±0.3^{c}	3.6±0.4^{d}	4.3±0.7^{abcd}
Kidney	0.7±0.1^{a}	0.7±0.0^{b}	0.7±0.1^{c}	0.7±0.0^{d}	0.8±0.2^{abcd}
Cecum	1.2±0.2	1.1±0.2	1.1±0.2	1.0±0.2^{a}	1.2±0.6^{a}
Adipose					
Epididymal	2.7±0.7	3.0±0.7	3.1±0.7	3.1±0.6	2.8±0.8
Perirenal	1.2±0.3^{abcd}	1.8±0.6^{a}	1.9±0.7^{b}	1.7±0.5^{c}	2.0±0.6^{d}
BAT	1.6±0.4^{abcd}	2.0±0.4^{a}	2.3±0.6^{b}	2.1±0.3^{c}	2.2±0.7^{d}
Total fat	5.5±1.2^{abcd}	6.8±1.1^{a}	7.3±1.1^{b}	6.9±1.0^{c}	6.9±1.8^{d}
Carcass	75±1^{abc}	74±1^{d}	74±1^{ae}	74±1^{bf}	72±2^{cdef}
**Body length (cm)**	13.0±0.4^{abc}	13.3±0.3	13.4±0.3^{a}	13.5±0.6^{b}	13.5±0.3^{c}
**BMI (kg/m^2)**	5.4±0.5^{ab}	5.4±0.4^{c}	5.6±0.4	5.7±0.5^{a}	5.8±0.3^{bc}
**Plasma**					
TC (mg/dl)	88±21^{a}	98±20	96±16	104±26	115±40^{a}
TG (mg/dl)	55±11^{ab}	68±19^{c}	66±22^{d}	75±20^{ae}	93±33^{bcde}

Values are mean±SD (n=13–14); Diet Code = the number of rats represented by each diet in the final group of Resistant or Susceptible, e.g. the number of 70:10, 60:20, 50:20, etc; CHO:fat Score = the 'recalculated' average CHO:Fat ratio based on the diets of rats included in the Diet Code groups; $^{a,b,c.}$ Means in a row sharing a common superscript differ ($p<0.05$) by by one-way ANOVA and Fisher's PLSD test.

Table S8. Diabetic assessment of 3 week old male Nile rats fed AFB diets with different CHO:Fat ratios and GLoads for 10 weeks sorted into Quintiles of Average kcal/day (Experiment 2) – Unsplit by T2DM.

	**Quintiles (kcal/day)**				
**Q_{kcal}**	**1**	**2**	**3**	**4**	**5**
**n=**	**13**	**14**	**14**	**14**	**14**
**kcal/day**	20.7±1.5	22.9±0.4	23.8±0.3	25.3±0.4	28.0±2.3
**P/S Ratio**	0.35	0.35	0.35	0.35	0.35
**Resistant/susceptible (% susceptible)** 10 week RBG <75mg/dl>	12/1 (8%)	11/3 (21%)	14/0 (0%)	6/8 (57%)	6/8 (57%)
10 week 30′ OGTT <175mg/dl>	11/2 (15%)	5/9 (64%)	10/4 (29%)	8/6 (43%)	3/11 (79%)
10 week FBG <60mg/dl>	10/3 (34%)	9/5 (36%)	12/2 (14%)	9/5 (36%)	8/6 (43%)
**Diet Code**	5, 3, 0, 5, 0	4, 2, 2, 3, 3	2, 3, 2, 5, 2	2, 3, 6, 0, 3	1, 3, 4, 0, 6
**CHO:fat Score**	3.8±2.8^{a}	3.0±2.8	2.3±2.2	2.4±2.1	1.8±1.8^{a}
**est CHO:fat:protein %energy**	63:17:20	60:20:20	56:24:20	56:24:20	51:29:20
**est. GLoad/2000 kcal**	227±55^{a}	196±76	191±64	198±68	166±78^{a}
**RBW (g)**					
Initial (3 weeks old)	29±4^{abc}	32±5^{a}	31±3	33±4^{b}	33±5^{c}
After 3 weeks	64±9^{ab}	68±7^{c}	70±7	73±7^{a}	74±8^{bc}
After 6 weeks	77±9^{abcd}	84±8^{ae}	86±8^{b}	89±9^{c}	91±9^{de}
After 10 weeks	89±13^{abc}	96±11^{d}	100±9^{a}	103±10^{b}	106±10^{cd}
**BW gain/day (g/day)**	0.8±0.1^{abc}	0.9±0.1	0.9±0.1^{a}	0.9±0.2^{b}	0.9±0.1^{c}
**Energy intake**					
g/day	11.7±1.4^{ab}	12.1±1.8^{c}	12.5±1.4	13.1±1.7^{a}	13.6±2.1^{bc}
**kcal/day**					
week 2	22.1±2.2^{abcd}	23.9±1.8a^{efg}	25.2±1.2b^{ehi}	26.7±1.5^{cfh}	28.0±1.9d^{gi}
week 8	20.9±2.1^{abcd}	22.8±1.4^{aef}	23.8±2.0^{bg}	25.1±1.6^{ceh}	29.9±3.2^{dfgh}
10 week average	20.7±1.5	22.9±0.4	23.8±0.3	25.3±0.4	28.0±2.3
**kcal/kg BW /10 week**	236±28^{a}	241±34^{b}	240±24^{c}	248±24	266±30^{abc}
**18:2(n-6) %energy**	3.1±1.8^{a}	4.1±2.5	4.3±2.1	4.1±2.2	5.1±2.5^{a}
**GLoad**					
/rat/day	2.4±0.6	2.2±0.9	2.3±0.8	2.5±0.9	2.3±1.1
/kg BW at 10weeks	27±7	23±8	23±8	24±8	24±8
Cumulative, 10 weeks	164±44	154±62	159±54	174±61	159±77
cumGLoad/rat/kcal	0.11^{a}	0.10	0.09	0.10	0.08^{a}
**10th Week Water Intake (ml)**	**32±10^{a}**	**30±6^{b}**	**33±12^{c}**	**34±15^{d}**	**60±45^{abcd}**
**Food efficiency (g BW gained/1000 kcal)**					
1-6 weeks	49±6^{a}	48±7	47±8	45±7	44±6^{a}
1-10 weeks	39±5^{a}	37±6	38±6^{b}	37±6	34±4^{ab}
**RBG (mg/dl)**					
After 6 weeks	64±9^{a}	61±9^{b}	64±18^{c}	83±39	113±120^{abc}
After 10 weeks	**82±68^{a}**	**64±15^{b}**	**60±7^{c}**	**122±102**	**191±174^{abc}**
Severity (>75mg/dl)	+7	-11	-15	+47	+116
**OGTT (BG mg/dl)**					

**After 6 weeks**					
FBG, 0 min	46±11	49±10	50±14	52±14	50±13
30 min	175±62^{a}	186±55^{b}	182±59^{c}	215±80	271±128^{abc}
**After 10 weeks**					
FBG, 0 min	47±14	53±18	55±23	52±15	62±27
30 min	136±46^{a}	178±79^{b}	164±55^{c}	187±72^{d}	294±138^{abcd}
60 min	86±16^{a}	140±69^{b}	117±69^{c}	129±77^{d}	254±169^{abcd}
**Organ weight (%BW)**					
Liver	3.1±0.3^{a}	3.3±0.3^{b}	3.4±0.3^{c}	3.5±0.4^{d}	4.0±0.8^{abcd}
Kidney	0.8±0.2	0.8±0.6	0.7±0.0	0.7±0.1	0.8±0.1
Cecum	1.1±0.2	1.1±0.3^{a}	1.1±0.2	1.0±0.3^{a}	1.1±0.3
Adipose					
Epididymal	2.4±0.7^{a}	3.0±0.9^{a}	2.6±0.5	2.9±0.7	2.7±0.5
Perirenal	1.3±0.5^{abcd}	1.7±0.5^{a}	1.6±0.4^{b}	1.7±0.4^{c}	1.7±0.4^{d}
BAT	1.7±0.6^{ab}	2.2±0.5^{a}	2.0±0.5	2.1±0.6	2.2±0.7^{b}
Total fat	5.4±1.6^{abc}	6.9±1.6^{a}	6.3±1.0	6.7±1.2^{b}	6.7±1.2^{c}
Carcass	76±2^{abcd}	74±2^{a}	74±2^{b}	74±2^{c}	73±1^{d}
**Body length (cm)**	12.9±0.5^{abc}	13.3±0.8^{a}	13.3±0.3	13.4±0.5^{b}	13.3±0.4^{c}
**BMI (kg/m^2)**	5.2±0.5^{ab}	5.3±0.6^{c}	5.5±0.4	5.5±0.6^{a}	5.7±0.4^{bc}
**Plasma**					
TC (mg/dl)	121±30	113±19	104±14^{a}	125±26^{a}	122±37
TG (mg/dl)	71±30^{ab}	89±26^{c}	75±17^{d}	108±48^{a}	128±76^{bcd}

Values are mean±SD (n=13–14); Diet Code = the number of rats represented by each diet in the final group of Resistant or Susceptible, e.g. the number of 70:10, 60:20, 50:20, etc; CHO:fat Score = the 'recalculated' average CHO:fat ratio based on the diets of rats included in the Diet Code groups; ^{a,b,c} Means in a row sharing a common superscript differ ($p<0.05$) by by one-way ANOVA and Fisher's PLSD test.

Table S9. Diabetic assessment of 3 week old male Nile rats fed PKOB diets with different CHO:fat ratios and GLoads for 10 weeks (Experiment 1) – sorted into Quintiles of Average kcal/day and split into resistant or susceptible to T2DM.

	**Quintiles (kcal/day)**									
**Q_{kcal}**	**1**		**2**		**3**		**4**		**5**	
**n=**	**13**		**14**		**14**		**14**		**14**	
**kcal/day**	**20.7±1.5**		**22.9±0.4**		**23.8±0.3**		**25.3±0.4**		**28.0±2.3**	
**P/S ratio**	1.00		1.00		1.00		1.00		1.00	
**Resistant/susceptible (%susceptible)**										
10 week RBG <75mg/dl>	11/2 (15%)		9/5 (36%)		8/6 (43%)		8/6 (43%)		4/10 (71%)	
10 week 30′OGTT <175mg/dl>	11/2 (15%)		9/5 (36%)		6/8 (57%)		4/10 (71%)		1/13	
10 week FBG <60mg/dl>	10/3 (30%)		10/4 (29%)		8/6 (43%)		11/3 (21%)		8/6 (43%)	
	**Data Split into *resistant* and *susceptible* rats**									
	**Resist**	**Suscept**	**Resist**	**Suscept**	**Resist**	**Suscept**	**Resist**	**Suscept**	**Resist**	**Suscept**
**n=**	**11**	**2**	**9**	**5**	**8**	**6**	**8**	**6**	**4**	**10**
**est CHO:fat:protein %energy**	**61:19:20**	**64:16:20**	**54:26:20**	**52:28:20**	**53:27:20**	**58:22:20**	**52:28:20**	**58:22:20**	**56:24:20**	**62:18:20**
**CHO:fat Score**	**3.2±3.0**	**4.0±4.2**	**2.1±2.1**	**1.9±2.9**	**2.0±2.4**	**2.7±2.4**	**1.9±2.3**	**2.7±0.8**	**2.3±3.2**	**3.5±2.4**
**Diet Code***	**4,0,1,6 0**	**1,0,0,1,0**	**1,2,2,2,2**	**1,0,0,2,2**	**1,2,1,0,4**	**1,2,1,1,1**	**1,1,2,1,3**	**0,5,0,1,0**	**1,0,1,0,2**	**3,2,5,0,0**
**GLoad/2000 kcal**	**209±58**	**221±83**	**185±70**	**154±81**	**163±89**	**203±72**	**168±79**	**231±34**	**162±97**	**235±35**
**RBW (g)**										
Initial (3 weeks of age)	31±4	29±2	32±6	31±4	30±4	32±4	31±3	32±4	35±3	33±6
After 3 weeks[c]	67±5	67±11	69±6	66±6	69±7	72±4	71±6	74±8	79±6	78±6
After 6 weeks[c]	79±7	83±18	85±8	83±6	89±6	92±7	91±3	94±9	100±8	95±6
After 10 weeks[c]	94±9	96±21	99±9	99±7	103±8	107±10	105±4	110±11	112±9	108±8
**BW gain/day (g/day)[cd]**	0.9±0.1	0.9±0.2	0.9±0.1	0.9±0.1	1.0±0.1	1.0±0.1	1.0±0.2	1.0±0.1	1.0±0.1	1.00±0.1
**Energy intake**										
g/day[c]	12.3±1.3	12.4±0.5	12.3±1.7	11.9±2.0	12.2±2.2	13.4±1.8	13.1±2.0	14.5±0.7	13.9±2.9	17.0±2.9
**kcal/d**										
week 2[c]	22.6±1.3	22.4±1.4	23.8±1.8	24.2±2.2	25.8±1.5	25.1±1.3	25.9±1.0	25.4±1.9	28.4±1.5	26.9±1.7
week 8[c]	21.8±1.6	22.0±2.4	23.4±1.1	23.5±1.1	24.8±2.4	25.1±1.2	27.3±2.2	25.1±1.1	29.3±2.6	29.1±3.5
10 week average[c]	22.3±1.0	22.0±2.0	24.1±0.3	24.1±0.3	25.0±0.4	25.2±0.3	26.5±0.7	26.2±0.5	28.4±1.0	30.4±3.4
**kcal/kg BW /10 week**	237±14	233±29	244±24	245±17	244±22	238±21	254±10	242±27	254±14	284±49
**18:2(n-6) %energy**	7.5±3.9	6.7±5.7	9.2±4.67	11.2±5.5	10.7±5.9	8.0±4.8	10.4±5.2	6.2±2.2	10.7±6.5	5.8±2.5
**GLoad**										
/rat/day	2.3±0.7	2.4±0.7	2.2±0.9	1.9±1.0	2.0±1.1	2.6±0.9	2.2±1.0	3.0±0.5	2.3±1.4	3.6±0.8
/kg BW at 10 weeks	25±7	26±13	23±9	19±10	20±11	24±9	21±11	28±6	21±14	34±10
Cumulative, 10 weeks	162±45	167±48	156±60	129±67	143±78	179±64	155±72	213±33	161±96	251±57
cumGLoad/rat/kcal	0.10	0.11	0.09	0.08	0.08	0.10	0.08	0.11	0.08	0.12
**10th week Water Intake (ml)**	31±7	19±3	36±10	33±16	35±14	42±15	41±9	65±55	42±16	172±180
**Food efficiency (g BWgained/1000 kcal)**										
1-6 weeks	49±8	51±11	46±6	46±10	50±5	48±6	47±2	48±7	47±4	46±9
1-10 weeks	38±4	40±7	37±6	38±4	39±4	39±6	38±6	39±4	37±3	34±7

**RBG (mg/dl)**										
After 6 weeks[c]	71±23	60±1	75±15	70±11	83±21	87±33	89±69	80±22	133±137	283±188
After 10 weeks[abc]	**61±11**	**86±4**	**59±9**	**193±107**	**62±7**	**111±49**	**62±6**	**196±113**	**59±8**	**346±167**
Severity (>75mg/dl)	-14	+11	-16	+118	-13	+36	-13	+121	-16	+271
**OGTT (BG mg/dl)**										
**After 6 weeks**										
FBG, 0 min	52±13	75±22	51±10	60±14	49±6	48±11	55±15	53±13	56±11	65±29
30 min[bc]	187±47	162±11	193±51	240±77	194±65	241±83	243±77	301±116	276±17	391±130
**After 10 weeks**										
FBG, 0 min	50±14	45±2	56±17	60±14	55±17	68±34	52±16	54±10	57±6	60±13
30 min[bc]	138±49	144±38	152±19	265±89	168±44	222±87	198±53	286±140	270±11	386±157
60 min[bc]	113±66	126±81	105±50	231±92	131±55	162±89	164±114	215±76	194±40	302±149
**Organ weight (%BW)**										
Liver[c]	3.3±0.3	3.4±0.3	3.5±0.3	3.6±0.4	4.3±0.7	3.3±0.3	3.4±0.3	3.5±0.3	3.6±0.4	4.3±0.7
Kidney	0.7±0.1	0.7±0.0	0.7±0.1	0.7±0.0	0.8±0.2	0.7±0.1	0.7±0.0	0.7±0.1	0.7±0.0	0.8±0.2
Cecum	1.2±0.2	1.1±0.2	1.1±0.2	1.0±0.2	1.2±0.6	1.2±0.3	1.1±0.2	1.1±0.2	1.0±0.2	1.2±0.6
Adipose										
Epididymal	2.7±0.7	3.0±0.7	3.1±0.7	3.1±0.6	2.8±0.8	2.7±0.8	3.0±0.8	3.1±0.5	3.1±0.4	2.8±0.9
Perirenal[ac]	1.2±0.3	1.8±0.6	1.9±0.7	1.7±0.5	2.0±0.6	1.2±0.3	1.8±0.6	1.9±0.7	1.7±0.5	2.0±0.6
BAT[c]	1.6±0.4	2.0±0.4	2.3±0.6	2.1±0.3	2.2±0.7	1.6±0.4	2.0±0.4	2.3±0.6	2.1±0.3	2.2±0.7
Total fat[ac]	5.5±1.2	6.8±1.1	7.3±1.1	6.9±1.0	6.9±1.8	5.5±1.2	6.8±1.1	7.3±1.1	6.9±1.0	6.9±1.8
Carcass[c]	75±1	74±1	74±1	74±1	72±2	75±1	74±1	74±1	74±1	72±2
**Body length (cm)**[cd]	13.0±0.4	13.3±0.3	13.4±0.3	13.5±0.6	13.5±0.3	13.0±0.4	13.3±0.4	13.4±0.3	13.5±0.6	13.5±0.3
**BMI (kg/m^2)**	5.4±0.5	5.4±0.4	5.6±0.4	5.7±0.5	5.8±0.3	5.4±0.5	5.4±0.4	5.6±0.5	5.7±0.5	5.8±0.3
**Plasma**										
TC (mg/dl)	90±21	80±27	98±13	98±28	90±9	103±20	95±20	116±31	103±13	119±46
TG (mg/dl)[bc]	56±10	50±17	62±17	78±21	63±17	71±28	67±13	86±24	73±20	100±35

Values are mean±SD (n=13–14); Diet Code = the number of rats represented by each diet in the final group of Resistant or Susceptible, e.g. The number of 70:10, 60:20, 50:20, etc; CHO:fat Score = the 'recalculated' average CHO:fat ratio based on the diets of rats included in the Diet Code groups; [a]Significant ($P < 0.05$) interaction term for quintile x diabetes class (RBG <75 mg/dl>) revealed by two-way ANOVA; [b]Significant ($P < 0.05$) effect of kcal/day by two-way ANOVA; [c]Significant ($P < 0.05$) increase due to diabetes (RBG >75 mg/dl) by two-way ANOVA; [d]Significant ($P < 0.05$) decrease due to diabetes (RBG >75 mg/dl) by two-way ANOVA.

Table S10. Diabetic assessment of 3 week old male Nile rats fed AFB diets with different CHO:fat ratios and GLoads for 10 weeks (Experiment 2) – sorted into Quintiles of Average kcal/day and split into resistant or susceptible to T2DM.

	**Quintiles (kcal/day)**									
**Q$_{kcal}$**	**1**		**2**		**3**		**4**		**5**	
**n=**	**13**		**14**		**14**		**14**		**14**	
Kcal/day	20.7±1.5		22.9±0.4		23.8±0.3		25.3±0.4		28.0±2.3	
P/S ratio	0.35		0.35		0.35		0.35		0.35	
**Resistant/susceptible (% susceptible)**										
10 week RBG <75mg/dl>	12/1 (8%)		11/3 (21%)		14/0 (0%)		6/8 (57%)		6/8 (57%)	
10 week 30′ OGTT <175mg/dl>	11/2 (15%)		5/9 (64%)		10/4 (29%)		8/6 (43%)		3/11 (79%)	
10 week FBG <60mg/dl>	10/3 (34%)		9/5 (36%)		12/2 (14%)		9/5 (36%)		8/6 (43%)	
	**Data Split into *resistant* and *susceptible* rats**									
	**resist**	**suscept**	**resist**	**suscept**	**resist**	**sus-**	**resist**	**suscept**	**resist**	**suscept**
**n=**	**12**	**1**	**11**	**3**	**14**	**0**	**6**	**8**	**6**	**8**
**est CHO:fat:protein %energy**	**62:18:20**	**70:10:20**	**62:18:20**	**36:44:20**	**56:24:20**	**n/a**	**44:36:20**	**62:18:20**	**40:40:20**	**56:24:20**
**CHO:fat Score**	**3.5±2.7**	**7**	**3.5±2.9**	**0.8±0.4**	**2.3±2.2**	**n/a**	**1.2±1.1**	**3.3±2.3**	**1.0±1.1**	**2.3±2.1**
**Diet Code***	**4,3,0,5,0**	**1,0,0,0,0**	**4,2,2,1,2**	**0,0,0,2,1**	**2,3,2,5,2**	**n/a**	**0,1,2,0,3**	**2,2,4,0,0**	**0,1,1,0,4**	**1,2,3,0,2**
**GLoad/2000 kcal**	**222±55**	**280**	**213±76**	**135±46**	**191±64**	**n/a**	**150±76**	**233±34**	**130±75**	**193±74**
**RBW (g)**										
Initial (3 weeks old)[b]	29±4	26	31±5	35±3	31±3	n/a	35±4	32±3	29±4	36±3
After 3 weeks[ab]	64±9	67	67±6	71±10	70±7	n/a	75±9	71±6	67±6	79±5
After 6 weeks[ab]	76±9	89	82±8	90±9	86±8	n/a	90±10	89±8	85±7	96±8
After 10 weeks[ab]	88±13	107	95±10	100±16	100±10	n/a	102±11	104±9	100±9	110±9
**BW gain/day (g/day)**	0.8±0.1	1.1	0.8±0.1	0.8±0.12	0.9±0.1	n/a	0.9±0.2	0.9±0.1	0.9±0.1	1.0±0.1
**Energy intake**										
g/day[c]	11.5±1.4	13.7	12.4±1.8	10.9±1.5	12.5±1.4	n/a	12.0±1.8	14.0±1.1	12.5±1.9	14.4±2.0
**kcal/day**										
week 2[abc]	21.9±2.2	24.4	23.8±1.8	24.0±2.3	25.2±1.2	n/a	26.7±1.1	26.8±1.8	27.9±2.2	28.1±1.7
week 8[abc]	20.8±2.2	21.6	22.5±1.4	23.8±0.1	23.8±2.0	n/a	25.4±1.5	25.0±1.8	30.8±2.9	29.3±3.5
10 week average[c]	20.5±1.5	22.1	22.9±0.4	22.8±0.4	23.8±0.3	n/a	25.4±0.4	25.2±0.4	27.8±2.9	28.0±2.0
**kcal/kg BW/day[bc]**	244±35	189	240±18	233±32	239±20	n/a	240±20	244±23	271±36	254±27
**18:2(n-6) %energy**	3.25±1.80	1.3	3.55±2.47	6.07±1.50	4.27±2.07	n/a	5.63±2.42	2.93±1.15	6.28±2.39	4.23±2.38
**GLoad**										
/rat/day	2.29±0.61	3.09	2.38±0.90	1.55±0.55	2.27±0.77	n/a	1.90±0.98	2.94±0.42	1.78±0.97	2.64±1.09
/kg BW at 10 weeks[a]	26±7	29	25±8	15±4	23±8	n/a	18±8	28±3	17±8	25±11
Cumulative, 10 weeks	160±43	216	167±63	109±38	159±54	n/a	133±69	206±29	124±68	185±77
cumGLoad/rat/day/	0.11	0.14	0.10	0.07	0.09	n/a	0.07	0.12	0.06	0.10
**10th Week Water Intake (ml)**	31±9	52	29±5	36±8	33±12	n/a	35±14	33±16	35±20	76±50

**Food efficiency (g BW gained/1000 kcal)**										
1-6 weeks	49±5	60	47±7	49±5	47±8	n/a	44±6	46±7	42±7	45±4
1-10 weeks	38±5	49	37±6	37±8	38±6	n/a	36±6	37±5	34±5	34±4
**RBG (mg/dl)**										
After 6 weeks[abc]	64±9	72	64±8	53±10	64±18	n/a	69±26	93±46	57±11	155±148
After 10 weeks[b]	**63±8**	**308**	**58±9**	**86±6**	**60±7**	**n/a**	**60±10**	**168±116**	**57±6**	**292±171**
Severity (>75mg/dl)	-12	+233	-17	+11	-15	n/a	-15	+93	-18	+217
**OGTT (BG mg/dl)**										
**After 6 weeks**										
FBG, 0 min	45±11	56	46±10	57±8	50±14	n/a	51±19	52±10	44±6	54±15
30 min[abc]	161±39	337	182±54	200±69	182±59	n/a	196±60	229±94	180±48	340±128
**After 10 weeks**										
FBG, 0 min[b]	47±14	40	48±17	68±18	55±23	n/a	47±16	55±14	49±11	72±33
30 min[abc]	127±42	211	166±75	220±96	164±55	n/a	156±47	211±81	184±34	377±127
60 min[abc]	88±15	67	121±37	209±124	117±69	n/a	122±74	134±84	116±55	357±149
**Organ weight (%BW)**										
Liver[abc]	3.1±0.2	3.5	3.4±0.2	3.1±0.2	3.4±0.3	n/a	3.3±0.2	3.6±0.4	3.5±0.3	4.4±0.8
Kidney	0.8±0.2	0.7	0.9±0.6	0.7±0.1	0.7±0.0	n/a	0.7±0.0	0.7±0.1	0.7±0.1	0.9±0.1
Cecum	1.1±0.2	1.1	1.1±0.2	1.3±0.5	1.1±0.2	n/a	1.0±0.5	1.0±0.1	1.2±0.3	1.1±0.3
Adipose										
Epididymal	2.4±0.7	2.6	2.7±0.9	3.7±0.8	2.6±0.5	n/a	2.8±0.2	2.9±0.9	2.7±0.6	2.7±0.4
Perirenal[b]	1.2±0.4	2.0	1.6±0.5	2.1±0.6	1.6±0.4	n/a	1.5±0.3	1.9±0.3	1.6±0.4	1.9±0.4
BAT[b]	1.6±0.5	2.7	2.2±0.5	2.3±0.7	2.0±0.5	n/a	1.9±0.7	2.3±0.5	2.0±0.7	2.4±0.6
Total fat[b]	5.2±1.6	7.2	6.5±1.7	8.1±0.5	6.3±1.0	n/a	6.2±1.0	7.1±1.1	6.2±1.3	7.0±1.2
Carcass[b]	76±2	72	74±2	73±2	74±2	n/a	74±1	74±2	74±1	73±1
**Body length (cm)[b]**	12.9±0.5	13.1	13.2±0.8	13.9±0.7	13.3±0.3	n/a	13.3±0.5	13.5±0.4	13.1±0.3	13.5±0.3
**BMI (kg/m^2)**	5.1±0.5	6.0	5.4±0.6	4.9±0.3	5.5±0.4	n/a	5.6±0.6	5.5±0.6	5.6±0.6	5.8±0.3
**Plasma**										
TC (mg/dl)	116±26	178	114±18	110±27	104±14	n/a	118±10	131±34	105±30	136±37
TG (mg/dl)[b]	64±19	146	89±28	88±24	75±17	n/a	83±26	128±53	74±32	168±75

Values are mean±SD (n=13–14); Diet Code = the number of rats represented by each diet in the final group of Resistant or Susceptible e.g. The number of 70:10, 60:20, 50:20, etc; CHO:fat Score = the 'recalculated' average CHO:fat ratio based on the diets of rats included in the Diet Code groups; [a]Significant (P < 0.05) interaction term for quintile x diabetes class (RBG <75 mg/dl>) revealed by two-way ANOVA; [b]Significant (P < 0.05) effect of kcal/day by two-way ANOVA; [c]Significant (P < 0.05) increase due to diabetes (RBG >75 mg/dl) by two-way ANOVA; [d]Significant (P < 0.05) decrease due to diabetes (RBG >75 mg/dl) by two-way ANOVA.

Table S11. Diabetic assessment of 3 week old male Nile rats fed PKOB diets with different CHO:fat ratios and GLoads for 10 weeks sorted into Quintiles of 10week RBG (Experiment 1).

	Quintiles (10 week RBG)				
**QRBG**	**1**	**2**	**3**	**4**	**5**
**n=**	**13**	**14**	**14**	**14**	**14**
	resist	resist	resist	suscept	suscept
**10week RBG**	51±6^{a}	62±1^{b}	70±3^{c}	101±40^{d}	353±110^{abcd}
**P/S Ratio**	1.00	1.00	1.00	1.00	1.00
**Resistant/Susceptible (%susceptible)**					
10 week RBG <75mg/dl>	13/0 (0%)	14/0 (0%)	13/1 (7%)	0/14 (100%)	0/14 (100%)
10 week 30′ OGTT <175mg/dl>	10/3 (23%)	8/6 (43%)	5/9 (64%)	7/7 (50%)	0/14 (100%)
10 week FBG <60mg/dl>	9/4 (29%)	12/2 (14%)	8/6 (43%)	9/5 (36%)	9/5 (36%)
**Diet Code**	3, 1, 2, 3, 4	1, 2, 3, 3, 5	4, 3, 2, 3, 2	3, 4, 2, 2, 3	3, 4, 4, 3, 0
**CHO:fat Score**	2.4±2.7	1.6±1.8	3.1±2.7	2.8±2.5	3.0±2.3
**est. CHO:fat:protein %energy**	56:24:20	49:31:20	60:20:20	59:21:20	60:20:20
**est. GLoad2000 kcal**	177±79	163±71^{a}	208±70	200±74	223±43^{a}
**RBW (g)**					
Initial (3 weeks of age)	30±5^{a}	31±3	33±4	31±4	33±5^{a}
After 3 weeks	70±5	69±8^{a}	70±6	71±7	75±7^{a}
After 6 weeks	87±6	86±11^{a}	87±8	89±10	93±8^{a}
After 10 weeks	103±8	100±11^{a}	101±9	103±11	108±9^{a}
**BW gain per day (g/day)**	0.9±0.1	0.9±0.1^{a}	0.9±0.2	0.9±0.2	1.0±0.1^{a}
**Energy intake**					
g/day	12.5±1.9^{a}	12.3±1.7^{b}	13.2±2.1^{c}	13.3±2.2^{d}	15.7±3.1^{abcd}
**kcal/day**					
week 2	25.0±1.9	25.2±2.9	24.3±2.5^{a}	25.6±2.0	26.8±2.7^{a}
week 8	25.4±1.5^{a}	25.9±2.9^{b}	25.4±2.7^{c}	25.3±2.4^{d}	31.5±6.7^{abcd}
10 week average	24.6±1.4^{a}	25.1±2.4^{b}	24.4±2.3^{c}	25.2±2.0^{d}	28.4±4.1^{abcd}
**kcal/kg BW /10 week**	103±8^{a}	100±11	101±9^{b}	103±11	108±9^{ab}
**18:2(n-6) %energy**	9.7±5.3	10.7±4.7^{a}	7.6±4.7	8.2±5.0	6.7±2.9^{a}
**GLoad**					
/rat/day	2.17±0.94^{a}	2.02±0.88^{b}	2.54±0.91	2.53±0.97	3.20±0.93^{ab}
/kg BW at 10 weeks	22±10^{a}	20±9^{b}	25±9	25±10	30±10^{ab}
Cumulative, 10 weeks	152±65^{a}	141±61^{b}	178±63	177±68	224±66^{ab}
cumGLoad/rat/kcal	0.09	0.08	0.10	0.10	0.11
**10th week Water Intake (ml)**	38±7^{a}	35±13^{b}	34±11^{c}	33±14^{d}	148±157^{abcd}
**Food efficiency (g BW gained/1000 kcal)**					
1-6 weeks	49±4	48±8	47±5	47±7	47±9
1-10 weeks	39±5	37±4	38±5	38±5	36±7
**RBG (mg/dl)**					
After 6 weeks	70±12^{a}	73±20^{b}	108±84^{c}	80±36^{d}	222±185^{abcd}
after 10 weeks	**51±6^{ab}**	**62±1^{c}**	**70±3^{d}**	**101±40^{ae}**	**353±110^{bcde}**
Severity (>75mg/dl)	-24	-13	-5	+26	+278
**OGTT (BG mg/dl)**					
**After 6 weeks**					
FBG, 0 min	51±9^{a}	51±9^{b}	54±15	55±15	64±25^{ab}
30 min	211±67^{a}	210±40^{b}	211±77^{c}	216±82^{d}	387±104^{abcd}

**After 10 weeks**					
FBG, 0 min	55±14	47±10^{ab}	58±17	59±24^{a}	61±11^{b}
30 min	154±56^{a}	174±50^{b}	192±57^{c}	193±88^{d}	400±108^{abcd}
60 min	134±73^{a}	127±48^{b}	141±97^{c}	158±83^{d}	308±111^{abcd}
**Organ weight (%BW)**					
Liver	3.4±0.4^{a}	3.6±0.4^{b}	3.4±0.4^{c}	3.5±0.4^{d}	4.2±0.7^{abcd}
Kidney	0.7±0.1^{a}	0.7±0.1^{b}	0.7±0.1^{c}	0.7±0.1^{d}	0.9±0.2^{abcd}
Cecum	1.1±0.2	1.0±0.1^{a}	1.1±0.2	1.0±0.2^{b}	1.3±0.5^{ab}
Adipose					
Epididymal	3.0±0.6	3.1±0.7	2.9±0.7	3.0±0.8	2.7±0.7
Perirenal	1.7±0.4	1.7±0.6	1.6±0.5	1.9±0.7	1.9±0.7
BAT	2.0±0.4	1.9±0.6	2.0±0.5	2.2±0.7	2.1±0.6
Total fat	6.6±1.0	6.7±1.5	6.5±1.3	7.1±1.4	6.7±1.7
Carcass	75±2^{a}	74±2	74±2^{b}	74±2	73±1^{ab}
**Body length (cm)**	13.3±0.2	13.2±0.4	13.5±0.7	13.3±0.4	13.4±0.3
**BMI (kg/m^2)**	5.6±0.4	5.5±0.4	5.4±0.5^{a}	5.6±0.4	5.8±0.4^{a}
**Plasma**					
TC (mg/dl)	100±19^{a}	90±15^{b}	93±15^{c}	96±22^{d}	123±41^{abcd}
TG (mg/dl)	64±17^{a}	57±12^{bc}	66±15^{d}	76±32^{be}	93±28^{acde}

Values are mean±SD (n=13–14); Diet Code = the number of rats represented by each diet in the final group of Resistant or Susceptible, e.g. the number of 70:10, 60:20, 50:20, etc; CHO:fat Score = the 'recalculated' average CHO:fat ratio based on the diets of rats included in the Diet Code groups; ^{a,b,c} Means in a row sharing a common superscript differ ($p<0.05$) by by one-way ANOVA and Fisher's PLSD test.

Table S12. Diabetic assessment of 3 week old male Nile rats fed AFB diets with different CHO:fat ratios and GLoads for 10 weeks sorted into Quintiles of 10week RBG (Experiment 2).

	**Quintiles (10 week RBG)**				
**QRBG**	**1**	**2**	**3**	**4**	**5**
**n=**	**13**	**14**	**14**	**14**	**14**
	**resist**	**resist**	**resist**	**suscept**	**suscept**
**10week RBG**	50±3[a]	57±2[b]	64±2[c]	76±5[d]	269±145[abcd]
P/S Ratio	**0.35**	**0.35**	**0.35**	**0.35**	**0.35**
**Resistant/susceptible (%susceptible)**					
10 week RBG <75mg/dl>	13/0 (0%)	14/0 (0%)	14/0 (0%)	7/7 (50%)	0/14 (100%)
10 week 30′ OGTT <175mg/dl>	11/2 (15%)	8/6 (43%)	8/6 (43%)	8/6 (43%)	2/12 (86%)
10 week FBG <60mg/dl>	11/2 (15%)	10/4 (29%)	10/4 (29%)	9/5 (36%)	7/7 (50%)
**Diet Code**	2, 2, 2, 2, 5	2, 6, 3, 2, 1	2, 1, 1, 6, 4	5,3,2,1,3	3, 2, 6, 2, 1
**CHO:fat Score**	2.1±2.4	2.8±2.0	1.9±2.3	3.5±2.8	2.8±2.4
**est. CHO:fat:protein %energy**	54:26:20	59:21:20	52:28:20	62:18:20	59:21:20
**est.GLoad/2000 kcal**	169±80	218±54[a]	165±69[a]	211±78	211±54
**RBW (g)**					
Initial (3 weeks of age)	30±5	30±3[a]	32±4	33±4	33±4[a]
After 3 weeks	65±9[ab]	67±7[c]	70±8	71±9[a]	75±6[bc]
After 6 weeks	81±11[a]	82±8[b]	86±8	87±11	92±7[ab]
After 10 weeks	91±14[ab]	95±12[c]	100±8	102±13[a]	106±8[bc]
**BW gain per day (g/day)**	0.8±0.2[a]	0.9±0.1	0.9±0.1	0.9±0.1	1.0±0.1[a]
**Energy intake**					
g/day	11.7±2.1[a]	12.7±1.2[b]	11.8±1.4[c]	12.8±1.8	14.0±1.6[abc]
**kcal/d**					
week 2	24.8±3.0[a]	24.7±3.1[b]	24.4±2.1[c]	25.0±2.3[d]	27.2±2.2[abcd]
week 8	23.2±4.2[a]	24.1±4.3	24.8±2.7	23.9±2.5[b]	26.7±4.1[ab]
10 week average	23.5±2.7[a]	23.7±3.5[b]	23.5±1.6[c]	23.9±2.0[d]	26.1±2.7[abcd]
**kcal/kg BW /10 week**	261±32[ab]	250±32	238±30[a]	236±23[b]	247±27
**18:2(n-6) %energy**	5.0±2.6	3.4±1.7[a]	5.1±2.2[a]	3.6±2.6	3.6±1.8
**Gload**					
/ rat / day	1.96±0.98[a]	2.52±0.55[b]	1.87±0.76[bcd]	2.49±0.88[c]	2.71±0.78[ad]
/ kg BW at 10 weeks	21±9	27±7[a]	19±8[ab]	25±9	26±8[b]
Cumulative, 10weeks	137±69[a]	177±38[b]	131±53[bcd]	174±62[c]	190±55[ad]
cumGload/rat/kcal	0.08	0.11	0.08	0.10	0.10
**10th Week Water Intake (ml)**	34±13[a]	33±14[b]	30±8[c]	28±6[d]	62±42[abcd]
**Food efficiency (g BW gained/1000 kcal)**					
1-6 weeks	43±8[a]	47±6	49±7[a]	47±6	47±7
1-10 weeks	35±6	37±6	39±5	38±5	37±6

**RBG (mg/dl)**					
After 6 weeks	62±13[a]	64±17[b]	66±18[c]	62±9[d]	132±116[abcd]
After 10weeks	**50±3[a]**	**57±2[b]**	**64±2[c]**	**76±5[d]**	**269±145[abcd]**
Severity	-25	-18	-11	+1	+194
**OGTT (BG mg/dl)**					
**After 6 weeks**					
FBG, 0 min	43±8[a]	49±10	49±16	50±15	55±8[a]
30 min	166±43[a]	167±51[b]	187±47[c]	194±64[d]	314±112[abcd]
**After 10 weeks**					
FBG, 0 min	47±11[a]	50±15[b]	50±25[c]	55±14	66±28[abc]
30 min	141±43[a]	162±54[b]	163±52[c]	169±70[d]	322±125[abcd]
60 min	93±38[a]	102±50[b]	128±63[c]	139±77[d]	264±166[abcd]
**Organ weight (%BW)**					
Liver	3.3±0.3[a]	3.3±0.3[b]	3.3±0.3[c]	3.4±0.2[d]	4.0±0.8[abcd]
Kidney	0.9±0.6[a]	0.7±0.0	0.7±0.1	0.7±0.0[a]	0.8±0.1
Cecum	1.0±0.3[a]	1.1±0.2	1.2±0.2[ab]	1.0±0.3[b]	1.1±0.3
Adipose					
Epididymal	2.5±0.8	2.4±0.5[abc]	2.9±0.5[a]	2.9±0.6[b]	2.9±0.7[c]
Perirenal	1.5±0.6[a]	1.4±0.4[bc]	1.6±0.4[d]	1.7±0.4[b]	2.0±0.3[acd]
BAT	1.9±0.6[ab]	1.9±0.5[cd]	1.9±0.5[e]	2.3±0.6[ac]	2.4±0.5[bde]
Total fat	5.9±1.9[ab]	5.6±1.2[cd]	6.4±1.3	6.9±1.1[ac]	7.3±0.9[bd]
Carcass	75±2[a]	75±2[b]	75±1[c]	74±2	73±1[abc]
**Body length (cm)**	12.9±0.3[abc]	13.1±0.4[d]	13.3±0.3[a]	13.4±0.7[b]	13.6±0.5[cd]
**BMI (kg/m^2)**	5.3±0.5	5.4±0.6	5.5±0.3	5.5±0.7	5.5±0.5
**Plasma**					
TC (mg/dl)	110±14[a]	115±23[b]	107±25[c]	115±15[d]	137±40[abcd]
TG (mg/dl)	68±23[a]	76±24[b]	77±25[c]	92±23[d]	157±66[abcd]

Values are mean±SD (n=13–14); Diet Code = the number of rats represented by each diet in the final group of Resistant or Susceptible, e.g. the number of 70:10, 60:20, 50:20, etc; CHO:fat Score = the 'recalculated' average CHO:fat ratio based on the diets of rats included in the Diet Code groups; [a,b,c] Means in a row sharing a common superscript differ ($p<0.05$) by by one-way ANOVA and Fisher's PLSD test.
